# Implicit solvent systematic coarse-graining of dioleoylphosphatidylethanolamine lipids: From the inverted hexagonal to the bilayer structure

**DOI:** 10.1371/journal.pone.0214673

**Published:** 2019-04-05

**Authors:** Saeed Mortezazadeh, Yousef Jamali, Hossein Naderi-Manesh, Alexander P. Lyubartsev

**Affiliations:** 1 Department of Biophysics, Faculty of Biological Sciences, Tarbiat Modares University, Tehran, Iran; 2 Department of Mathematics, Tarbiat Modares University, Tehran, Iran; 3 School of Biological Sciences, Institute for Research in Fundamental Sciences (IPM), Tehran, Iran; 4 Department of Materials and Environmental Chemistry, Arrhenius Laboratory, Stockholm University, Stockholm, Sweden; University of Calgary, CANADA

## Abstract

Lamellar and hexagonal lipid structures are of particular importance in the biological processes such as membrane fusion and budding. Atomistic simulations of formation of these phases and transitions between them are computationally prohibitive, hence development of coarse-grained models is an important part of the methodological development in this area. Here we apply systematic bottom-up coarse-graining to model different phase structures formed by 1,2-dioleoylphosphatidylethanolamine (DOPE) lipid molecules. We started from atomistic simulations of DOPE lipids in water carried out at two different water/lipid molar ratio corresponding to the lamellar L_*α*_ and inverted hexagonal H_II_ structures at low and high lipid concentrations respectively. The atomistic trajectories were mapped to coarse-grained trajectories, in which each lipid was represented by 14 coarse-grained sites. Then the inverse Monte Carlo method was used to compute the effective coarse-grained potentials which for the coarse-grain model reproduce the same structural properties as the atomistic simulations. The potentials derived from the low concentration atomistic simulation were only able to form a bilayer structure, while both L_*α*_ and H_II_ lipid phases were formed in simulations with potentials obtained at high concentration. The typical atomistic configurations of lipids at high concentration combine fragments of both lamellar and non-lamellar structures, that is reflected in the extracted coarse-grained potentials which become transferable and can form a wide range of structures including the inverted hexagonal, bilayer, tubule, vesicle and micellar structures.

## Introduction

Phospholipids are the main ingredients of the cell membranes that separate cells from their surrounding. In addition to their key roles in biology, phospholipids are considered as a suitable candidate for nanocarriers in drug delivery systems in pharmaceutical applications [1, 2]. This is due to their capability to form a wide range of self-assembled structures including lamellar (bilayers), spherical (micelles and liposomes), tubular (hexagonal phases), and other more complicated structures such as cubic phases. Two important factors are involved in the formation and phase transition of the lipid self-assembled structures. The first factor lies in the shape and chemical properties of lipid molecules. The amphiphilic property of phospholipids is the principle cause of their self-assembly in aqueous solutions. Also, the ratio of the size of the lipid head to its tail (depending on the chemical nature of the head, length of the chains, number and place of the unsaturated hydrocarbon bonds) induces a specific packing factor affecting the self-assembled structures which can be formed. The second factor is the thermodynamic properties of the sample such as temperature and hydration (or concentration). Molecular modeling, taking into account all these factors, could be a valuable tool to investigate the mechanism of the self-assembly of lipid molecules.

The typical system sizes needed to model lipid self-assembly are too large to study them with all-atom molecular dynamics (MD) simulations. Therefore, to tackle this limitation, one needs to reduce the number of degrees of freedom by neglecting unimportant details and thus speed up the computations. This approach is called Coarse-Graining (CG) [3] which includes two basic strategies known as bottom-up and top-down. The bottom-up systematic coarse-graining uses atomistic simulations to derive effective potential functions for coarse-grained sites. This approach can be implemented using several methods such as the Iterative Boltzmann Inversion [4], Inverse Monte Carlo [5], force matching [6], and relative entropy [7]. The first two cases are considered as structure-based methods due to use of the pair correlation functions as a reference to build the effective potentials. The force matching method uses forces acting on a CG particle as a fitting property. The relative entropy method minimize the entropy difference between the atomistic and CG descriptions, and it was shown to be equivalent to the structure-based methods as it reconstructs pair distribution functions [8]. There are several good literature reviews on the systematics coarse-graining methods covering various methodological issues [9–11]. The main aim of the top-down approach is to reproduce key experimental data, such as partitioning of solutes between polar and apolar solvents. The Martini model [12] is the most famous model of this type which exploits the standard form of molecular mechanical force field with Lennard-Jones and electrostatic potential energy functions for non-bonded interactions and harmonic potential functions for bond and angle interactions. Both approaches have their own advantages and limitations which can be very generally summarized that the bottom-up approach is better suitable to capture specific details of the interactions between the involved molecules whereas the top-down approach provides a force field framework that usually can be easily extended to other systems [13].

Both top-down and bottom-up approaches were used to study lipid self-assembly. Models developed using the top-down approach, such as the Martini [14] and ELBA [15] models, have been used to study the formation of various lipid phases. A common feature of these models is the use of explicit solvent (water). In the Martini model, each solvent particle is equivalent to 4 atomistic water molecules while in the ELBA model each water molecule is represented by a CG particle. Models developed using systematic coarse-graining often use implicit water description which save computational time. Shelly et al [16] used IBI method to obtain CG potentials with the explicit solvent model of one particle per water molecule. Voth and colleagues have developed an implicit solvent model for a mixture of several lipids using the force matching method [17, 18]. The force matching was also used in papers [19] to simulate membrane proteins in physiologically representative mixed bilayers. Self-assembly of lipids into bilayer and vesicle structures within implicit solvent CG models derived by the IMC approach was described in papers [20, 21]. In a number of other bottom-up solvent-free models, hydrophobic behavior of hydrocarbon groups has been corrected by introducing an empirically parametrized additional attractive interaction [22, 23].

Phospholipids exhibit different phase behavior from inverse micelle to vesicle and micellar structures upon increase of the water content [24]. Among various self-assembled structures of the phospholipids, lamellar and inverted hexagonal phases are of particular importance in the formation of local domains within the membranes that lead to certain functions [25]. The phase transition involving these phases plays a key role in the balancing of lamellar/non-lamellar structures associated with the cell membrane processes. In this work we investigated phase behaviour of 1,2-dioleoylphosphatidylethanolamine (DOPE) lipids, which form inverted hexagonal (H_II_) phase at low water content and bilayer (L_*α*_) structures [26, 27] at high water content. For this purpose we developed a solvent-free CG model of DOPE lipid using systematic structure-based coarse-graining. The model was used to simulate formation of both H_II_ and L_*α*_ lipid phases. Since the bottom-up approaches are known to be dependent on the thermodynamic state of the system, atomistic simulations of DOPE molecules were performed under two different water contents corresponding to conditions of the lamellar and inverted hexagonal phases.

## Methods

### Theoretical background

Systematic coarse-graining includes two main steps: the CG molecular mapping design and computation of CG interaction potential functions. The coarse-grain mapping can be done in different ways depending on the chosen level of resolution suitable to describe the studied phenomena, and importance of specific chemical details. When a CG mapping scheme is determined, the next step is to define effective interaction potentials between the CG sites. The CG potential function usually includes bonded and non-bonded terms as well as coulombic interactions. The initial guess for each bonded and non-bonded terms can be obtained by direct Boltzmann inversion [4]:

$$\begin{array}{cc} U(r,\vartheta ,\varphi ) & =U_{bond}\left(r\right)+U_{angle}\left(\vartheta \right)+U_{torsion}\left(\varphi \right)\\ & \;+U_{non-bond}\left(r\right)+U_{coulomb}\\ U(r) & =-k_{B}T\text{ln}\frac{\left\langle S(r)\right\rangle }{4\pi r^{2}}\\ U(\vartheta ) & =-k_{B}T\text{ln}\frac{\left\langle S(\vartheta )\right\rangle }{\text{sin}(\vartheta )}\\ U(\varphi ) & =-k_{B}T\text{ln}\left\langle S\left(\varphi \right)\right\rangle \end{array}$$ (1)

where *k*_*B*_ and *T* are the Boltzmann constant and temperature respectively. The average histograms of bonded and non-bonded distance distributions 〈*S*(*r*)〉, angle 〈*S*(*ϑ*)〉, and torsion angle 〈*S*(*φ*)〉 distributions are calculated from the mapped atomistic trajectory. However, the initial potential functions (1) usually cannot reproduce the reference atomistic distribution functions. A method to improve the CG potentials is the Iterative Boltzmann Inversion (IBI) [10] which refines them during multiple iterative simulations. The tabulated potentials at the end of the *n*th CG simulation are modified according to the following equation:

$$U_{\alpha }^{n+1}=U_{\alpha }^{n}+{\Delta U}_{\alpha }^{n}=U_{\alpha }^{n}+ak_{B}T\text{ln}\frac{\left\langle S_{\alpha }^{n}\right\rangle }{\left\langle S_{\alpha }^{ref}\right\rangle }$$ (2)

where *a*, 0 < *a* < 1 is a correction factor to regulate the convergence of the distribution functions. The iterative process proceeds until an acceptable convergence is observed between the atomistic reference $\langle S_{\alpha }^{ref}\rangle$ and CG simulation $\langle S_{\alpha }^{n}\rangle$ distribution functions.

The basic assumptions of the IBI is that all terms of the tabulated potentials are independent of each other. This assumption leads often to convergence problem in multi-component systems, which include many types of different non-bonded interactions. In the Inverse Monte Carlo method, the CG potential functions are corrected by taking into account correlations between all the interactions. Details of this method can be found in papers [5, 28]. Within IMC, the CG potentials are modified by inverting the correlation matrix based on the following equation:

$$\begin{array}{cc} \Delta U_{\alpha }^{n} & =a\sum_{\gamma }A_{\alpha \gamma }^{-1}\times \left(\left\langle S_{\alpha }^{ref}\right\rangle -\left\langle S_{\alpha }^{n}\right\rangle \right)\\ A_{\alpha \gamma } & =\frac{\left(\left\langle S_{\alpha }^{n}\right\rangle \left\langle S_{\gamma }^{n}\right\rangle -\left\langle S_{\alpha }^{n}S_{\gamma }^{n}\right\rangle \right)}{k_{B}T} \end{array}$$ (3)

The correlation matrix (*A*_*α*γ_) is calculated between all terms of bonded and non-bonded interactions. This is usually done by Monte Carlo sampling, however, any sampling algorithm, such as constant-temperature molecular dynamics simulation, can be used provided that the phase space canonical distribution is generated.

### Atomistic simulations

Atomistic simulations of DOPE molecules in water were performed using Gromacs 5.0.7 software [29] at two different lipid/water molar ratios. Similar to the setup of previous work [21], 60 DOPE molecules were simulated in two different states named here LC (low concentration) and HC (high concentration). In the LC system, 60 lipid molecules were dissolved in 2000 water molecules (equivalent to 44.6 wt% water content), while the HC system consisted of 60 lipid molecules and 540 molecules of water, which is equivalent to 18 wt% water content. Since the total charge of each lipid molecule is zero, there was no need to add ions to neutralize the box. The initial state of LC system was prepared by random (uniform) distribution of lipids in the cubic periodic simulation box and filling the remaining space by water. In the HC system, the initial state was prepared by setting the lipids in a pseudo-inverted hexagonal phase, which was done by a short simulation of lipids in a triclinic box with unit cell angles (90°, 90°, 120°), and subsequent change of the geometry to a cubic cell. Before starting the simulation, 20000 steps of energy minimization were performed using the steepest descent method for both LC and HC systems. Then molecular dynamics simulations were carried out for 500 ns. The Slipids force field [30] was used for DOPE molecules together with TIP3P [31] model for water. The bonds were constrained using LINCS algorithm which allowed 2 fs time step. The NPT ensemble with temperature 303 K and pressure 1 bar was maintained by the V-rescale thermostat [32] with the time constant 0.5 ps and Parrinello-Rahman [33] barostat with the time constant 5 ps. Electrostatic interactions were treated by the fast smooth Particle-Mesh Ewald (PME) method [34] with 1.2 nm cutoff radius. Also, the force switching method [35] with the inner cutoff radius of 0.8 nm and the outer radius of 1.2 nm was used to calculate the Lennard-Jones interactions. The first 100 ns of the simulations were considered as equilibration, while the rest of the trajectory was used to calculate the reference distribution functions for building coarse-grain models. The VMD.1.9.2 [36] and Packmol softwares [37] were used for visualization and generation of the initial structures respectively.

Gromacs script files for simulating the LC and HC systems can be found in S1 Data.

### Coarse-grained model

#### Mapping scheme

**Fig 1:**
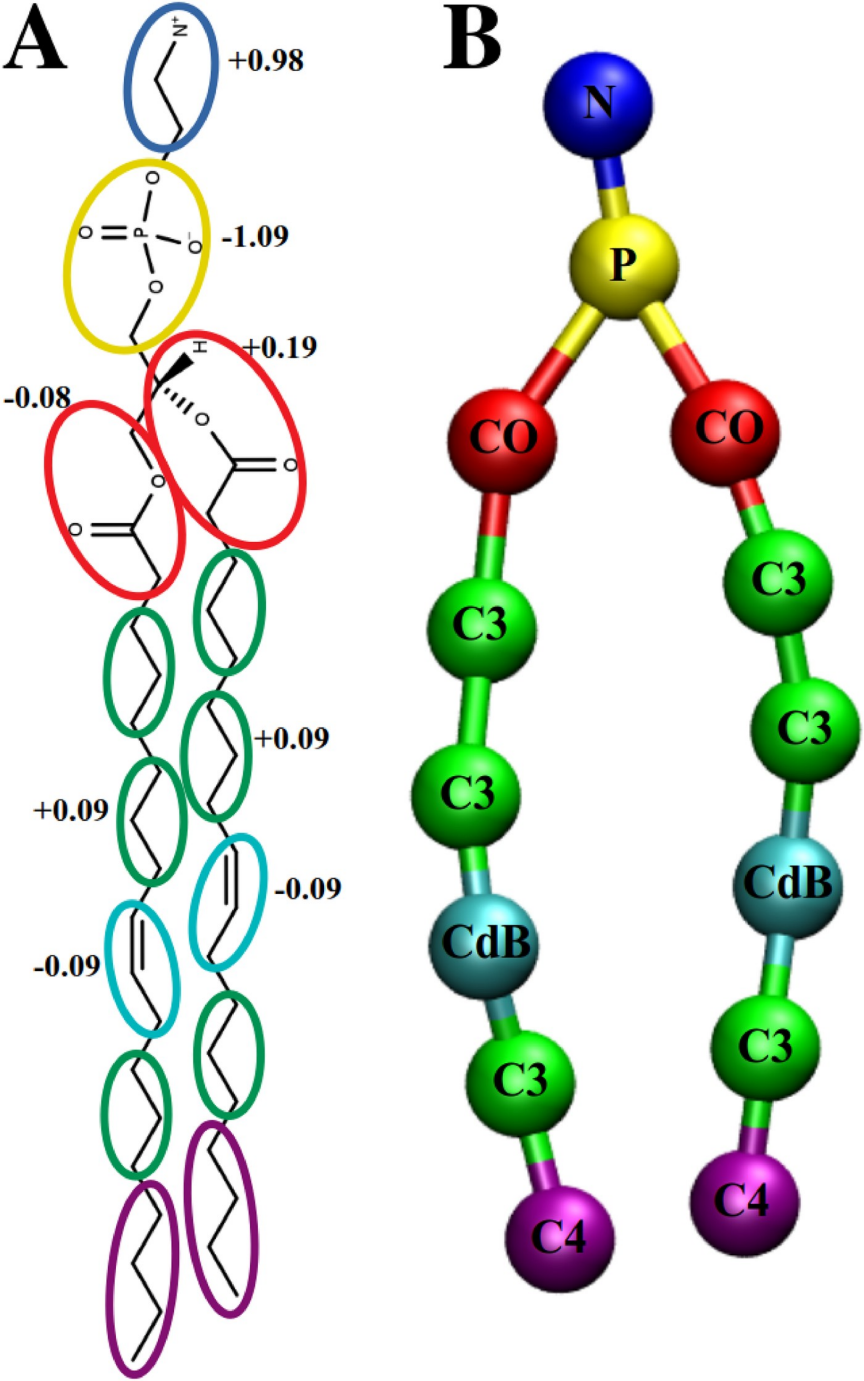
Mapping scheme of the DOPE molecule. (A) Atomistic representation of DOPE molecule showing atom groups constituting each CG site. Each type of CG site is shown in a separate color: blue bead—amine group, yellow bead—phosphate group, red beads—ester groups, green beads—propane groups, cyan beads—propene groups, and purple beads—butane groups. Non-zero charge of each CG site is shown next to it. (B) CG representation of DOPE molecule. Names of the CG sites as well as bonding between sites are shown.

The first step in the coarse-graining is specification of a mapping scheme to determine the beads of the CG model from the atomistic representation. In this study, water molecules were removed from the CG model and each DOPE molecule containing 129 atoms was mapped to a CG molecule with 14 beads as shown in Fig 1. There are six bead types in the CG model of DOPE, each of them representing a distinct chemical group: N (choline group), P (phosphate group), CO (ester group with a part of glycerol moiety), C3 (a fragment of three saturated hydrocarbons), CdB (a fragment of three hydrocarbons containing one double bond), and C4 (a chain of 4 saturated hydrocarbons). The position of each coarse-grain bead was determined as the center of mass of the corresponding atomistic group. The partial charge of each CG bead was calculated as a sum of the partial atom charges of the atoms constituting the given bead. Bonded interactions between the DOPE CG beads consisted of six types of bond pairwise potentials: N-P, P-CO, CO-C3, C3-C3, C3-CdB, and C3-C4, 7 types of angle potentials: N-P-CO, P-CO-C3, CO-P-CO, CO-C3-C3, C3-C3-CdB, C3-CdB-C3, and CdB-C3-C4, and 6 types of torsion angle potentials: N-P-CO-C3, P-CO-C3-C3, CO-P-CO-C3, CO-C3-C3-CdB, C3-C3-CdB-C3, and C3-CdB-C3-C4. Furthermore, there are 21 types of non-bonded pairwise interactions between 6 different bead types. Within the molecule, non-bonded interactions were set to zero for any atom pair involved in the bonded interactions. To compute effective potentials representing the listed above interactions in the CG model, reference distribution functions between corresponding CG sites were computed after mapping of the atomistic trajectory to its coarse-grained representation.

#### Coarse-grained simulations

In this work we used LAMMPS-2017 software [38] both as a sampling engine in the IBI/IMC computations of the CG potentials, and in subsequent simulations of the CG systems at larger scale. The software is equipped with GPU package [39, 40], which allows us to run high-performance simulation for systems described by tabulated potentials. Since in the CG model water molecules were considered implicitly, the water content of the CG lipid systems was determined by the density of lipids in the simulation box. The conjugate gradient method was used for energy minimization before each CG simulation. Langevin dynamics [41] with time constant 0.5 ps and time step 10 fs was used for sampling of the phase space. Note that dynamics in solvent-free CG models is artificially accelerated, by this reason simulation time in CG simulations is often scaled to relate it to the real time. In this work we report unscaled simulation time since we do not specifically study dynamical properties, but use Langevin dynamics to sample the configurational space.

Preparation of the potential tables for LAMMPS software was done as follows: each table was smoothed through radial basis function interpolation. Torsion angle potentials were interpolated periodically while potential tables of bond, angle, and left-hand side of the non-bonded interactions were extrapolated by a quadratic function. The right-hand side of the non-bonded potentials was extrapolated by polynomial functions so that the potential and force at the cutoff radius became zero. The cutoff radius of the short-range non-bonded potentials was set to 2 nm. To treat tabulated potentials efficiently, the linear spline interpolation was applied in the LAMMPS scripts. In addition to the non-bonded short-range potential determined within the IBI/IMC procedure, the CG beads interacted by the electrostatic interactions due to charges on beads, scaled by the value of dielectric permittivity *ϵ* = 78. The electrostatic interactions were treated by the PME method. Such separation of electrostatic and short-range interactions in solvent-mediated CG potentials was validated in previous works [42, 43].

#### Computation of the coarse-grained potentials by IBI/IMC

Initial potentials were calculated from the direct Boltzmann inversion (1) of the reference distribution functions and IBI method (2) was used in the beginning of the iterative process. After that the CG potentials were further refined by the IMC equations (3). We performed 20 IBI iterations followed by 20 IMC iterations to obtain CG potentials reproducing reference RDFs within the uncertainty of the simulations. The simulation time of each iteration in the IBI process was 25 ns with a sampling interval of 2.5 ps after 2 ns of equilibration. For the IMC process, simulation and equilibration times were 50 ns and 4 ns respectively during the first 10 iterations and doubled in the next 10 iterations. Increase of the simulation time on the later iterations is necessary to reduce the statistical error in calculating the correlation matrix. The resulting CG potentials at the end of the IMC process were used in the further CG simulations. The inverse procedure was performed for both LC and HC system resulting in two different sets of CG potentials. All steps of the systematic coarse-graining process including mapping the atomistic trajectory to CG trajectory, generating the CG topology file, calculating distribution functions and refining the CG potentials through IBI/IMC methods were performed by a home code written in python language.

## Results and discussions

### Atomistic simulations

**Fig 2:**
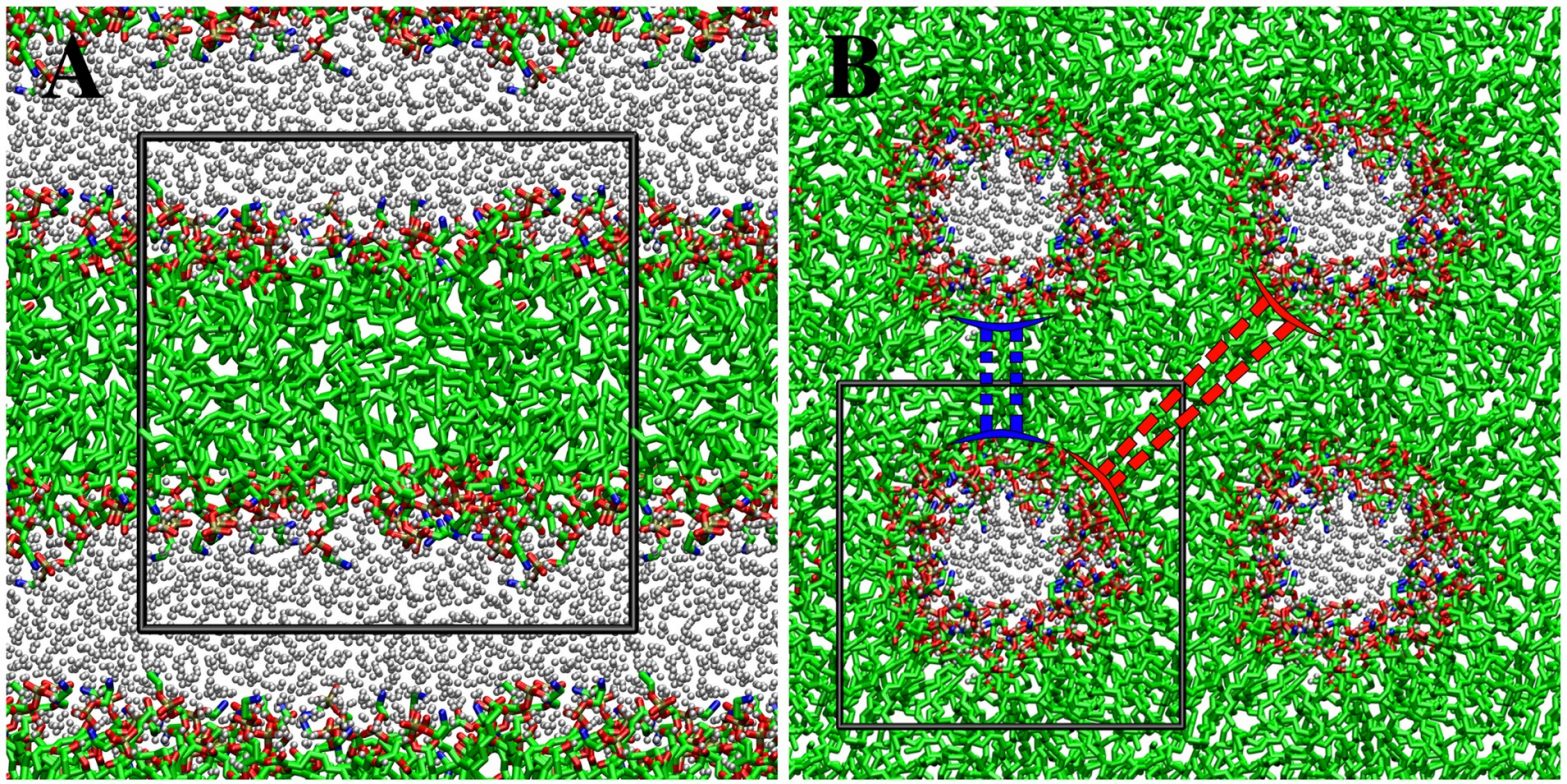
Structures obtained from the atomistic simulation of LC and HC states. (A) bilayer structure of the LC state. (B) pseudo-inverted hexagonal structure of the HC state. The blue and red dashed lines indicate lamellar and non-lamellar structural features respectively. Hydrogen atoms are not shown.

The final structures of the atomistic simulation of LC and HC systems are shown in Fig 2. In the LC system the lipids formed a lipid bilayer structure. In the HC system, the initial pseudo-inverted hexagonal structure was generally kept during the whole simulation. However, the final structure of the HC system showed characteristics of both lamellar and non-lamellar structures simultaneously (Fig 2(B)). The lipid density evaluated from the average box size was found to be 0.45 *nm*^−3^ and 0.67 *nm*^−3^ in the LC and HC systems respectively. These densities were used in calculations of the CG potentials which were carried out in the NVT ensemble.

### Convergence of the IBI/IMC procedure

The last 400 ns of the atomistic trajectories of LC and HC systems were mapped to the CG representations which were used to compute reference distribution functions for the IBI/IMC procedure. During the IBI/IMC procedure, convergence of the computed distribution functions to their reference values at each iteration was controlled by the total mean square deviations for all distribution functions:

$$\delta^{n}=\sum_{I=1}^{N_{I}}\sum_{\alpha =1}^{N_{\alpha }}\frac{1}{l_{\alpha }N_{\alpha }}{\left(\left\langle S_{\alpha }^{ref}\right\rangle -\left\langle S_{\alpha }^{n}\right\rangle \right)}^{2}$$ (4)

**Fig 3:**
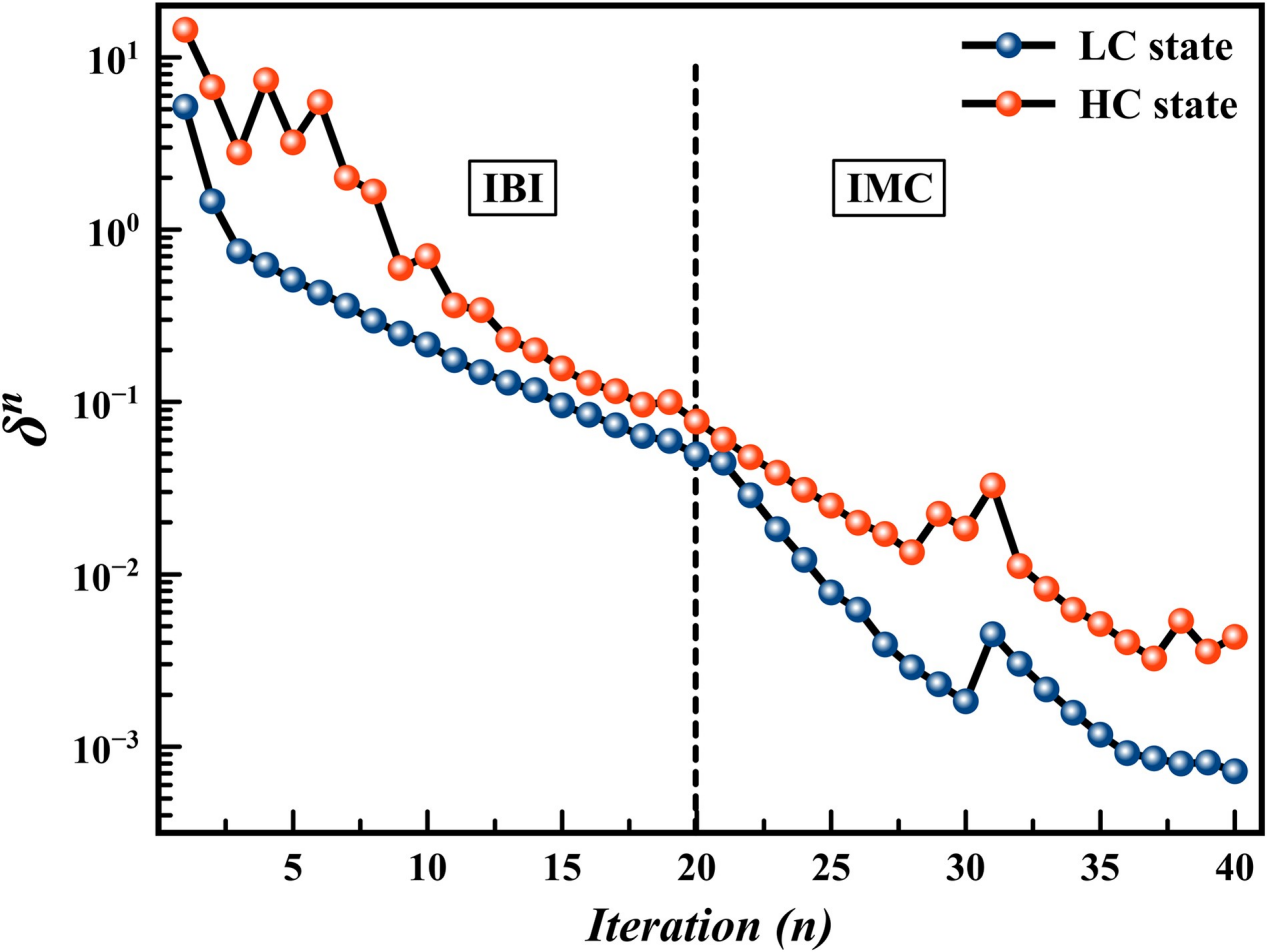
Convergence of IBI/IMC process. Total deviation of the simulated CG distribution functions from their reference values during the iterative processes.

In Eq (4), *N*_*I*_ is the total number of bonded and non-bonded interactions. *N*_*α*_ and *l*_*α*_ are the number of bin and bin width of each distribution function respectively. Fig 3 shows the deviation from the reference distribution function (*δ*^*n*^) during the 40 steps of the IBI/IMC process. Deviation in the HC model is greater than the LC model, however the convergence was acceptable at the end of 40 iterations for both models. The deviation in the IMC process was decreasing more rapidly than in the IBI process, which indicates the advantage of using IMC for fine-tuning of the CG potentials. However, using the IBI method is advantageous on the initial stages of the potential fitting process. The relative increase in deviation at iterations 31 for both models is due to reinitiation of a new series of IMC iterations from a random mixture as a starting point (for other iterations the last configuration of the preceding iteration was used as a starting point).

**Fig 4:**
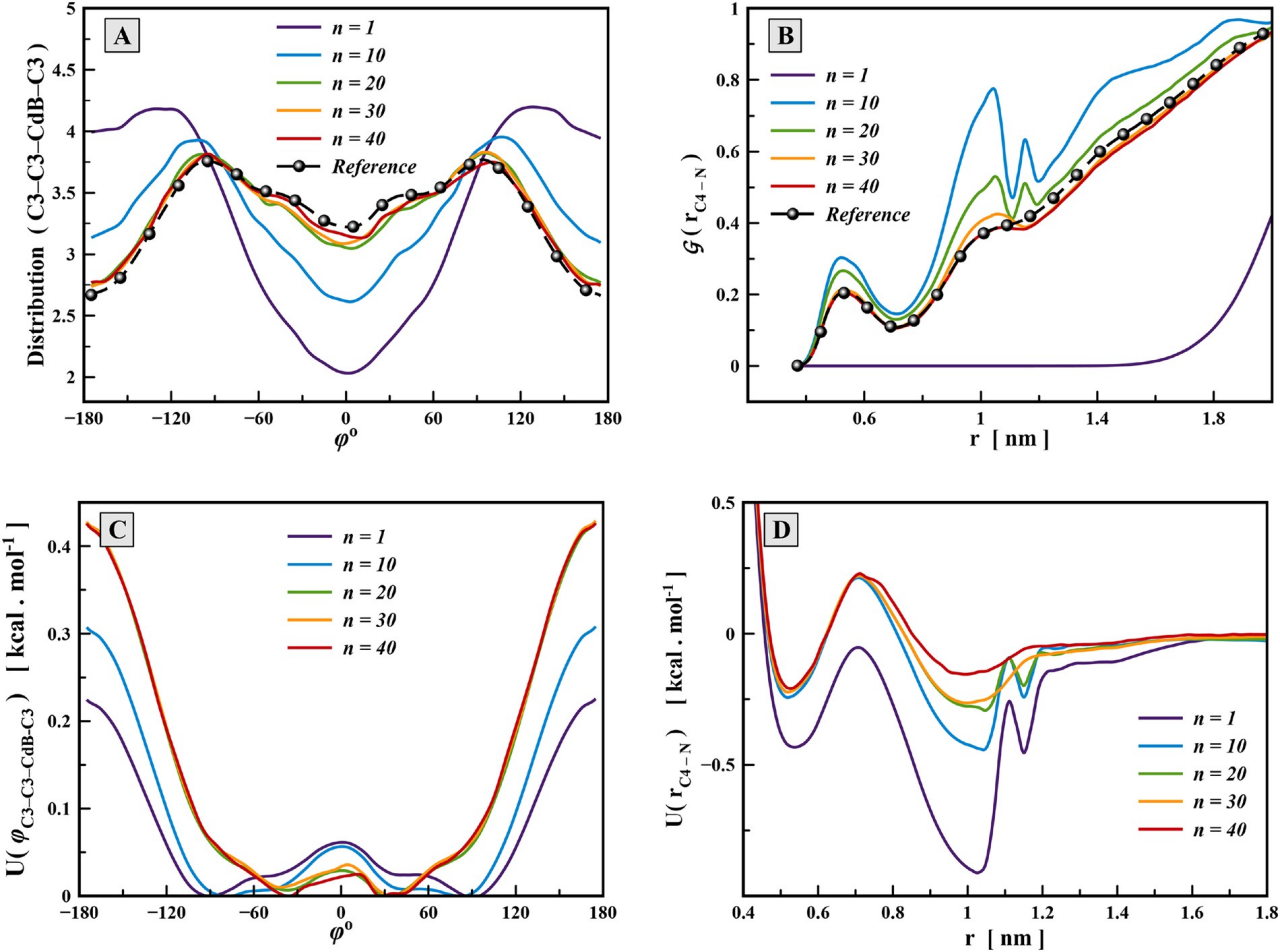
Two examples of convergence of CG potentials and distribution functions during the iterative process. (A) and (C) convergence of the distribution function (A) and CG potential (C) for torsion angle interaction C3-C3-CdB-C3. (B) and (D) convergence of the distribution function (B) and CG potential (D) for non-bonded interaction C4-N.

Fig 4 shows two instances of distribution functions and corresponding potentials during the iterative process, one for a torsion interaction and the other for non-bonded C4-N interaction. The C3-CdB-C3-C3 torsion potential controls the rotation around C3-CdB bond in the middle of lipid chains. Significant changes in the potential function compared to the result of direct Boltzmann inversion (*n* = 1) are clearly seen. Potential energy wells have been shifted to ±40° during the potential correction process to reproduce maximum of the distribution functions at ±116°. Also, the energy barrier at ±180° was roughly doubled preventing too easy rotation around the C3-CdB bond. These substantial changes of the CG torsion potential to fit distribution of the atomistic model demonstrate importance of taking into account torsion potentials in lipid CG models. The major part of the torsion potential changes was observed during the IBI process whereas the IMC process finally tuned the torsion angle potential to provide complete convergence of the distribution function. Similar picture was observed for other torsion angle interactions (for more information please see S1 Fig). While refinement of the direct Boltzmann inversion is clearly needed for torsion angle potentials, correction of the bond and angle potentials was negligible. This indicates that for bond and angle interactions use of direct Boltzmann Inversion may be sufficient. In general we can conclude that IBI potential refinement is sufficient to produce all terms of bonded interactions.

Convergence of the radial distribution function and non-bonded potential between bead types C4 and N in the HC model is shown in Fig 4(B) and 4(D). During 20 steps of the IBI process, the main features of the reference distribution have been reproduced in some extent. However some artificial peaks appeared in the range of distances 1—1.2 nm which do not correspond to any feature of the reference radial distribution function. These extra peaks appear because the IBI method assumes that all types of interaction are independent of each other, and it corrects the potential functions disregarding correlations between them. However, updating any bin value of the potential tables affects not only distribution function of the same bin, but also affects others. Similar effects of poor convergence were seen during the IBI process for some other non-bonded potentials including C4-N, C4-P, CdB-N and CdB-P, in both HC and LC models. All these types of the non-bond potentials are related to different interactions between the head, center, and tails of the lipid molecules which are strongly interconnected with each other.

When IMC refinement of the CG potentials was turned on, during 20 iterations the artificial features of the non-bonded C4-N potential were completely eliminated and the resulting pairwise potential adopted a physically meaningful form with two wells reflecting contact and next-neighbour interactions, and smoothly going to zero at distances above 1.2 nm. Unlike bonded potentials, the use of IMC method is necessary for refinement of the non-bonded interactions in complex multi-site molecular systems.

A complete set of the final CG potentials for the LC and HC models in a tabulated form can be found in S1 Data.

### Comparison of LC and HC models

**Fig 5:**
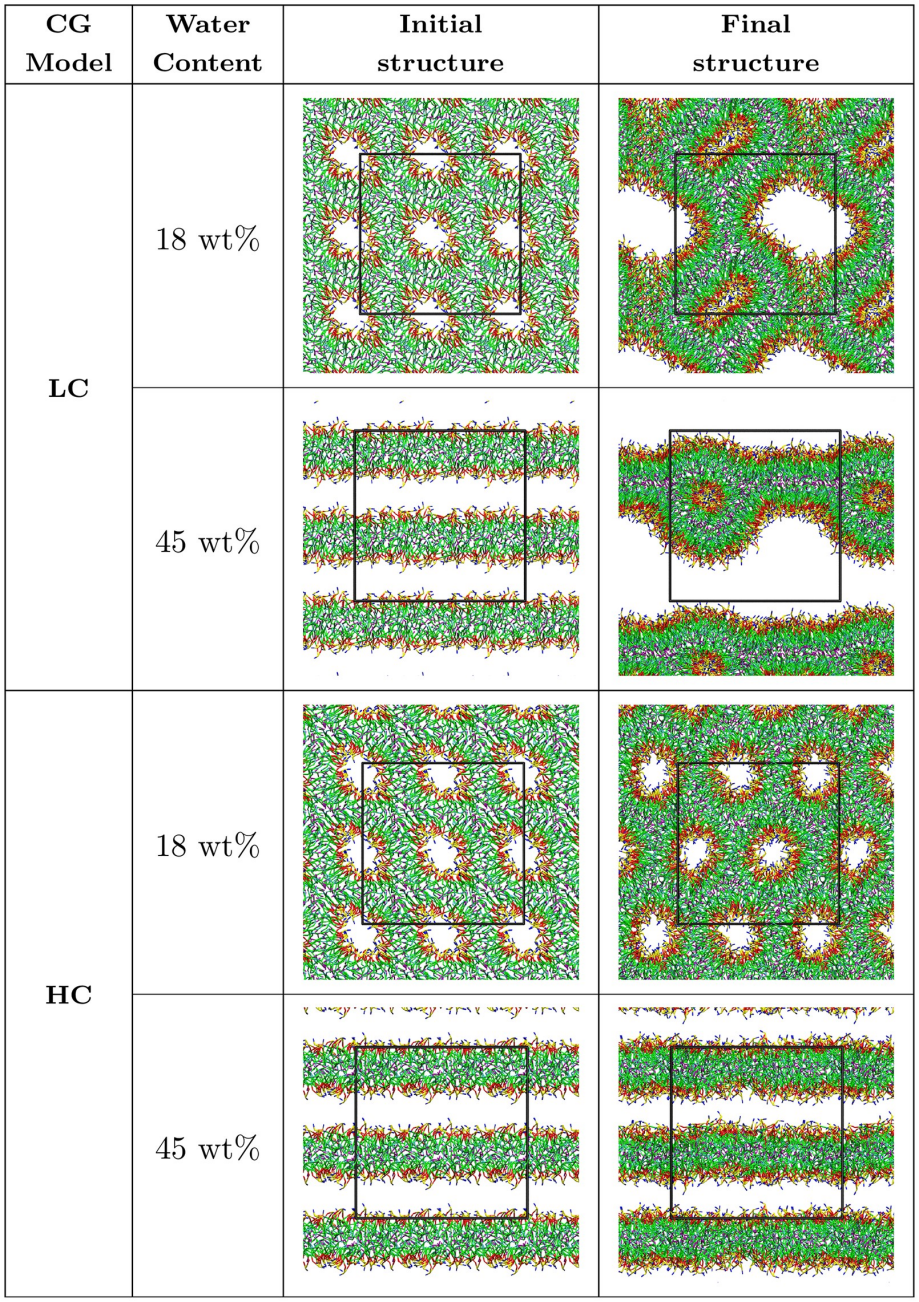
Initial and final snapshots of the 40 ns CG simulations of 480 lipid molecules at two different water contents by using potential functions of the LC and HC models.

To test the bottom-up derived LC and HC models, several simulations were performed with 480 CG lipid molecules. Fig 5 shows the initial and final states of 40 ns simulation of DOPE lipids at 18 wt% and 45 wt% water contents. In order to determine the ability of both LC and HC models to stabilize the L_*α*_ and H_II_ lipid phases, the initial simulation structures for 18 wt% and 45 wt% water content were taken according to the pseudo-inverted hexagonal and bilayer structures respectively. In case of the LC model (with CG potentials obtained from low-concentration atomistic simulations) both simulations resulted in a bilayer phase. In 18 wt% water content, the initial state representing inverted hexagonal cylinders was unstable and final lamellar structure with bilayer fragments forming “boxes” was obtained, that indicates the inability of the LC model to stabilize the H_II_ phase of the DOPE lipids at high lipid densities. In 45 wt% water content, the initial lipid multilayer structure fused together resulting in the formation of a lipidic particle structure. The lipidic particle is an intermediate structure when lamellar structures are fused [44]. Opposite to the LC model, the HC model stabilized both the lamellar and non-lamellar lipid phases, and a clear pattern of the inverted hexagonal structure was formed during the simulation at 18 wt% water content. The results of Fig 5 confirm the ability of the HC model to stabilize both L_*α*_ and H_II_ lipid phases at the appropriate lipid concentrations.

**Fig 6:**
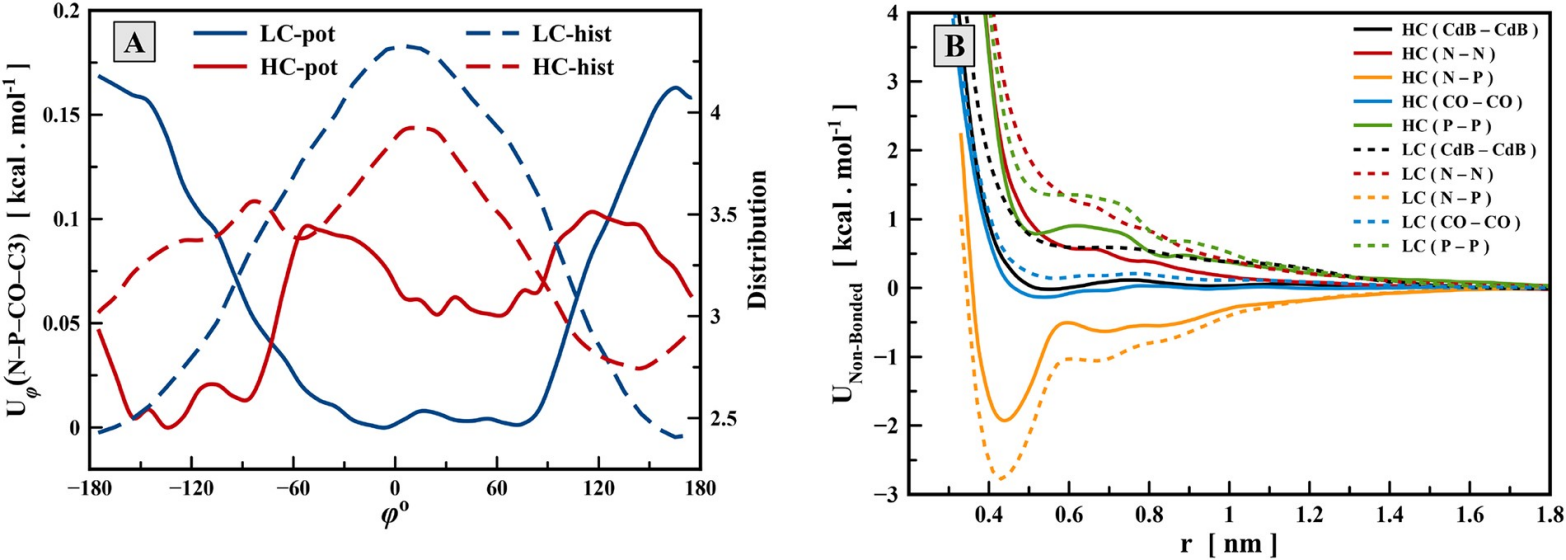
Comparison of LC and HC model. (A) The torsion potential and torsion angle distribution function of N-P-CO-C3 torsional angle. (B) THe pair-wise non-bonded potentials and corresponding radial distribution functions for five selected non-bonded interactions that shows the greatest difference between the LC and HC models.

Since the two bottom-up derived CG models showed qualitatively different results, it is instructive to analyze which features of the CG potentials are responsible for such behaviour. The comparison showed that non-bonded potentials in the LC and HC models are similar in shape with the same number and positions of maxima and minima, while only depths of potential wells are different (S2 Fig). Among all bonded potentials, there is only one torsion angle potential, N-P-CO-C3, which differs noticeably between HC and LC models, as shown in Fig 6A. This torsion potential controls rotation around the bond of the phosphate-ester groups at the lipid head, which is responsible for orientation of the dipole N-P vector relative to the lipid tails. As it can be seen, the potential function does not directly corresponds (via direct Boltzmann inversion) to the reference torsion distribution for both models. The HC potential function has two energy wells. The first is in the range of −160° to −90° and the latter is in the range of 0° to + 80° (note that the asymmetry of this torsion potential is related to the chiral nature of the lipid glycerol moiety). Although maximum of the distribution function in the HC model is located in the second range, the first well of the potential function is deeper than the second one. By comparison with the potential function of the LC model, it can be concluded that these two potential wells of the HC model are related to the inverted hexagonal and bilayer structures respectively, elements of both of which were observed in the atomistic HC simulations. In contrast, the energy barriers of the LC potential at torsional angles around ±180° function may hinder the formation of favorable torsion angles for the inverted hexagonal structure. This barrier appeared in the LC model because in the underlying atomistic simulations the lipids were arranged mostly in the lammelar bilayer structure where the corresponding range of N-P-CO-C3 torsion angles was poorly populated.

Fig 6B shows five non-bonded potential functions which have the greatest difference between the LC and HC models. These interactions are CdB-CdB, N-N, N-P, CO-CO and P-P, which are related to the interactions of the heads and chains of lipid molecules. Only the N-P interaction has a strong attractive part of the potential energy while others are repulsive. The balance in the attractive/repulsive forces of lipid head is originated from the decrease in the attractive force of N-P interaction as well as the decrease in the repulsive force of other interactions. Similar changes of the non-bonded CG interactions with concentration were observed previously for DMPC lipids [21]. These changes in the non-bond potential functions, being of the order of 1 kcal/mol, can certainly affect conditions for stabilization of the inverted hexagonal or bilayer structures in the CG model.

To investigate further the role of the bonded and non-bonded potentials in the formation of various phases of the DOPE molecules, mixed models combining bonded and non-bonded potential functions of the LC and HC models were constructed. The initial and final structures of the simulations using various combinations of the LC/HC potentials are presented in S3 Fig. The $\left(U_{Bonded}^{HC}+U_{Non-Bonded}^{LC}\right)$ potential functions were unable to stabilize the hexagonal structure, while the correct structure was obtained using the $\left(U_{Bonded}^{LC}+U_{Non-Bonded}^{HC}\right)$ potential functions with the pseudo-inverted hexagonal structure as the starting point. However, the $\left(U_{Bonded}^{LC}+U_{Non-Bonded}^{HC}\right)$ potential functions were unable to form the exact shape of the inverted hexagonal structure starting from a random mixture. These results showed that the non-bonded potential functions play a key role in the aggregation of lipid molecules as well as in the formation of a general structure, whereas the torsion angle potentials play an important role in the fine-tuning structural details of the inverted hexagonal phase. Therefore, both the bonded and non-bonded potential functions of the HC model are necessary to stabilize both of the L_*α*_ and H_II_ lipid phases at the corresponding concentrations.

### Formation of the L_*α*_ and H_II_ lipid phases

**Fig 7:**
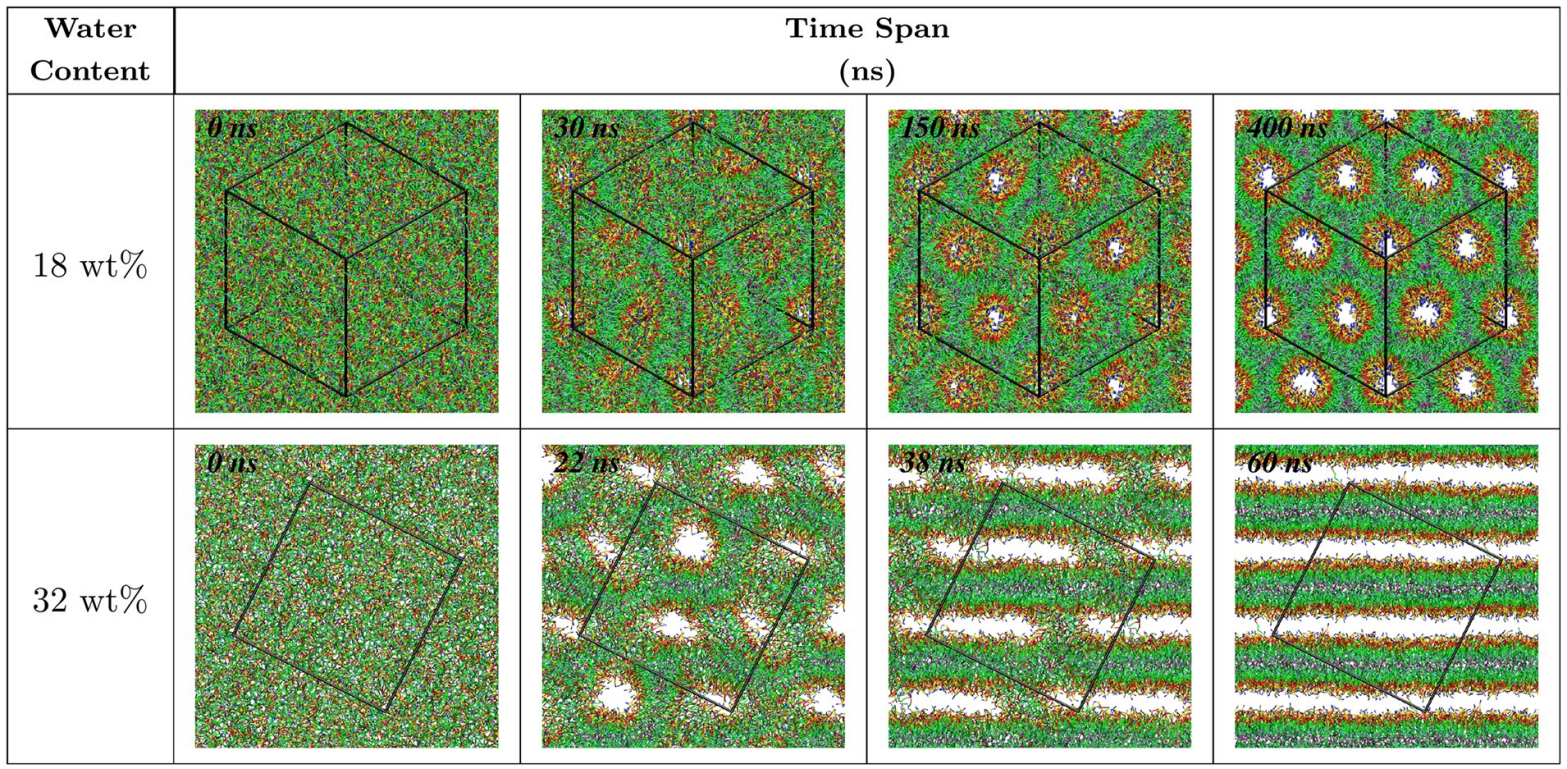
Snapshots of CG simulations of 1000 lipid molecules at two different water contents using the HC model showing formation of L_*α*_ and H_II_ lipid phases.

A crucial test of CG potentials is to examine their ability to form correct lipid phases at different water contents. For this purpose we carried out simulations of 1000 CG DOPE lipid molecules using potential functions of the HC model at various water contents, starting from a random structure. The structures formed during simulations at 18 wt% and 32 wt% water contents are shown in Fig 7. At both densities, tubular structures appeared after about 25 ns of the simulations. These structures were first formed in several parts of the box in 18 wt% water content. With a time, hexagonal structures were gradually developing along the diagonal of the box (S1 Video). This happened likely because such orientation of cylinders allows to accommodate a hexagonal structure in a periodic cubic cell. The exchange of lipid molecules between formed cylinders continued until the equilibrium was reached with completion of almost ideal H_II_ phase. In 32 wt% water content, local tubular structures were not stable and quickly converted to the disk-like structures which further fused together and formed the L_*α*_ lipid phase (S2 Video). During this process, the stalk-like structure was clearly observed around 38 ns of simulation. This structure is known as an intermediate state in the H_II_ − L_*α*_ phase transition [45].

**Fig 8:**
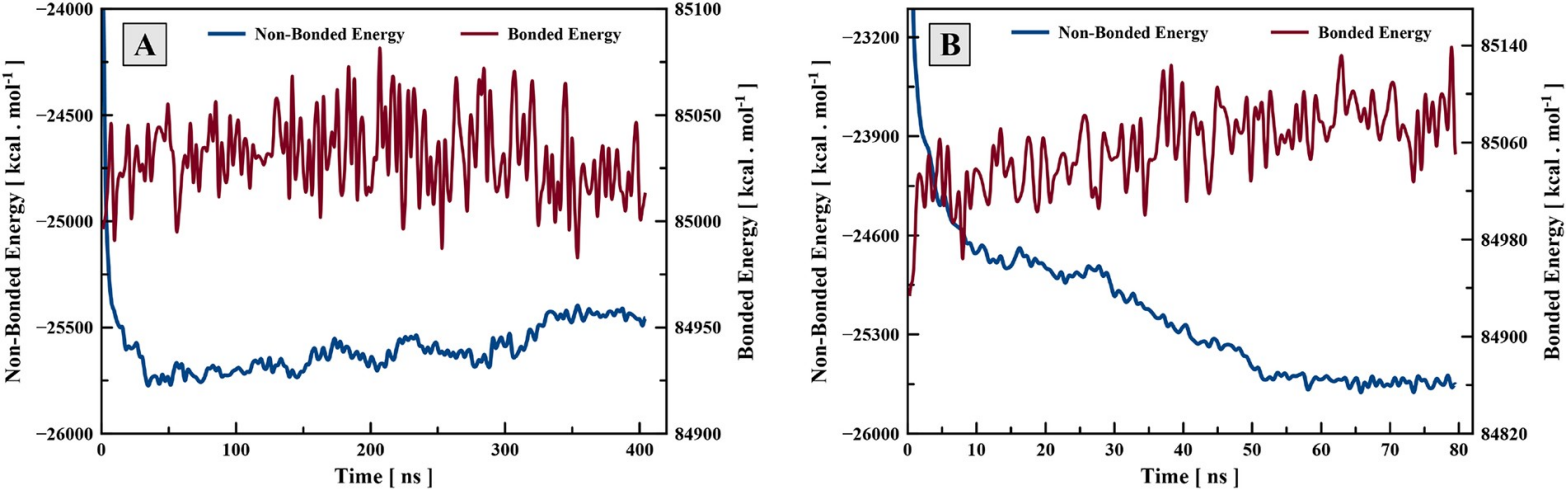
Bonded and non-bonded potential energies of the system of 1000 CG lipid molecules during the simulation. (A) System at 18 wt% water content during formation of the inverted hexagonal structure; (B) System at 32 wt% water content during formation of the bilayer structure.

The energy of lipid molecules showed different behavior during the formation of lipid phases. Fig 8 shows the bonded and non-bonded energies of lipid molecules at 18 wt% and 32 wt% water contents over time. In 18 wt% water content, an increase in the non-bonded potential energy was seen after 50 ns of the simulation which coincide in time with the development of the hexagonal structure. After 350 ns, torsion angle interactions caused the hexagonal structure to be stabilized which led to a reduction in bonded potential energy. An overall increase of the potential energy during 50-300 ns period of the simulation means that formation of H_II_ phase is entropy-driven, which is in agreement with experimental observations that H_II_ phase is stabilized with increase of temperature [26]. At 32 wt% water content, instability of the initial tubular structures is associated with an increase in bonded potential energy and a decrease in the non-bonded potential energy. In the both cases, the bonded potential functions (in particular the torsion angle interactions) play an important role in the fixation of the final inverted hexagonal and bilayer structures at the equilibrium point. The formation and stabilization of both L_*α*_ and H_II_ phases of the DOPE molecule at different water contents indicate transferability of the potentials derived within the HC model.

**Fig 9:**
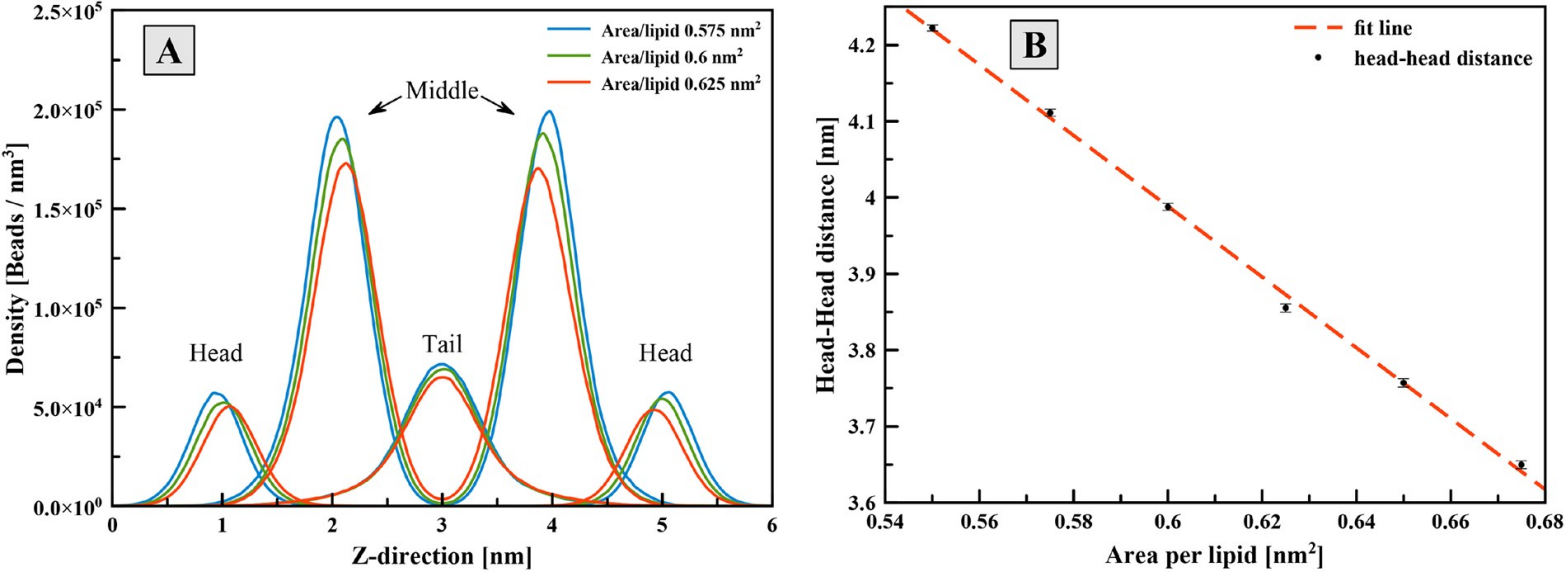
A) Density profile of different parts of CG lipid molecules across bilayer; B) the membrane thickness at various values of area per lipid.

The area per lipid (*A*_*L*_) is considered as one of the essential properties of the lipid bilayer structures. The *A*_*L*_ and lipid thickness are indicators whether or not the correct bilayer structure is formed. For this purpose, a bilayer structure consisting of 100 CG lipid molecules was simulated at various *A*_*L*_ for 100 ns. The lipid thickness was extracted from the z-density profile of the lipid particles as shown in Fig 9(A). The density profile of the lipid head group was calculated from the histogram of the z-projections of the N and P beads relative to the bilayer center. The overlap in the distribution functions of different lipid regions indicates the fluidity of the lipid bilayer structure. The thickness of the bilayer decreases with increasing *A*_*L*_ as shown in Fig 9(B). Particularly, the lipid thickness was 3.988 ± 0.005 nm at 0.6 nm^2^ lipid per area. The *A*_*L*_ and membrane thickness of bilayer structure of the DOPE molecule have been reported 0.604 ± 0.005 nm^2^ and 3.93 nm, using the atomistic simulation [30]. Also, experimental data for *A*_*L*_ have been reported 0.6 nm^2^ [46], which are in a good agreement with the results of the present study.

**Fig 10:**
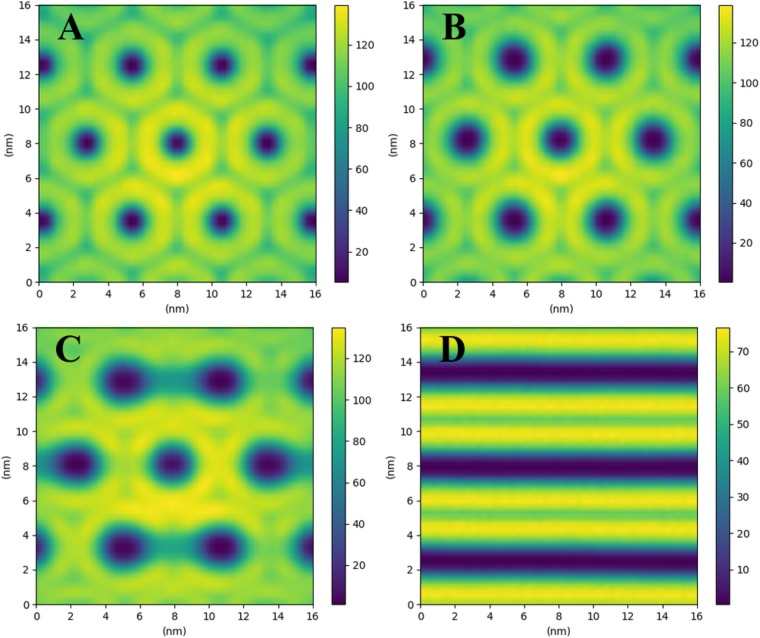
The cross-sectional 2D histogram of the systems of 1000 CG lipid molecules taken perpendicularly to the structures formed (see Fig 7) at various water contents of (A) 12 wt%, (B) 18 wt%, (C) 26 wt%, (D) 32 wt%. The central cylinder going through diagonal of the box has the largest length, which makes it looking more colorful.

To determine the water content corresponding to the phase transition between H_II_ and L_*α*_ phases, systems containing 1000 lipid molecules were simulated at different densities for 200 ns. Two-dimensional histograms of the cross-section of the structures at various densities (in the plane perpendicular to the direction of cylinders seen in Fig 7) are shown in Fig 10. The expansion of the hexagonal structure and the increase in the diameter of the hollow cylinders with increase of the water content from 12 wt% to 26 wt% is clearly seen. The separation distance between the cylinders is increasing from 5.2 nm (12 wt%) to 5.8 nm (26 wt%), this results is consistent with simulations of the inverted hexagonal phase of DOPE lipids within MARTINI model where spacing between the cylinders was found between 4.8 nm and 6.7 nm for the range of 8 wt% to 28 wt% water content and 308K temperature [14]. In our work the H_II_ − L_*α*_ phase transition was observed at 26 wt% water content. In this case, more than half of the molecules were found in the inverted hexagonal structure while other half was tending to form the lamellar structure. Above this value, bilayer and lamellar structures were observed. Experimental studies have reported the formation of inverted hexagonal structures below 32 wt% water content at temperature 300 K [26, 27] which is in a good agreement with 26 wt% water content at the transition point of our study.

**Fig 11:**
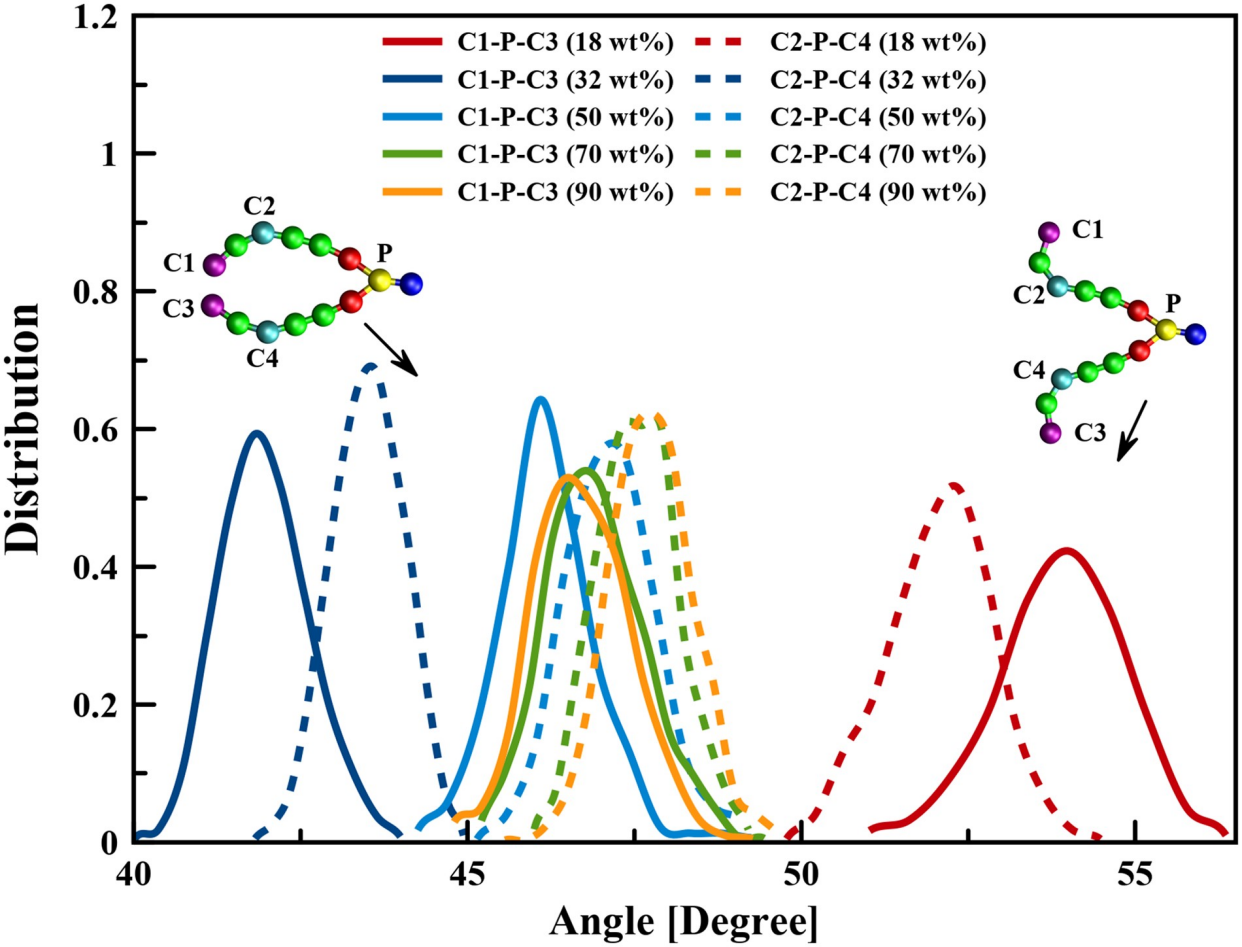
The distribution functions of the two order parameters representing the angles $\hat{\text{C}1-\text{P}-\text{C}3}$ and $\hat{\text{C}2-\text{P}-\text{C}4}$. The molecular shape of the DOPE at 18 wt% water content is different from the other water contents.

The final structures of simulations at 50 wt%, 70 wt%, and 90 wt% water contents are shown in S4 Fig. With increasing the box volume, the effects of periodic boundary conditions were effectively eliminated and tubular and unilamellar vesicle structures were obtained in the 50 wt% and 70 wt% water contents. In 90 wt% water content several aggregated structures including spherical, elliptical, and rod-like micelles were observed. It is however probable that in such a big box equilibration was not reached during the simulation time. Nevertheless we analyzed appearance of different aggregated structures by calculations of two “order parameters” which discriminate between lamellar and non-lamellar phases. One order parameters is determined as angle between vectors directed from P site to the middle of the two tails (to CdB beads), while the other is the angle between P site to the ends of lipid tails. These parameters represent the effective cone angle of the DOPE molecules that shown in Fig 11 for the lamellar and non-lamellar structures. By increasing the water content, the conical angle of each lipid molecule is reduced and the lipid molecules appear longer and approaching to a “cylindrical” shape. These angles has the highest value in the hexagonal structure and the lowest value in the bilayer structure. In other non-lamellar structures the conical angle is typically between these two values. By comparison the distribution function of these two order parameters at different concentrations (Fig 11), one can deduce that at high water content the lipids adopt structures intermediate between the lamellar and inverse hexagonal phases.

### Comparison with top-down CG models

At present, Martini force field is likely the most widely used coarse-grained model to simulate lipids and lipid structures [12]. This force field was also developed to simulate other biological molecules [47]. Each solvent particle in this model has been composed of 4 atomistic water molecules so that the explicit solvent model is necessary for stabilization of various lipid phases. Also, electrostatic interactions are included into this model since the phosphate and amine group of lipids have explicit electric charges. Stability of the inverted hexagonal structure has been reported in simulations using Martini model in the range of 8 wt% to 28 wt% water content [14]. Also, the formation of a hexagonal structure starting with a bilayer structure has been observed. The spontaneous formation of a hexagonal structure from a random mixture was reported only for small systems in high water contents [12]. According to Marrink and Mark, “Larger systems or systems at lower hydration levels easily get trapped into various kinds of metastable intermediate states, making the method less practical for predicting relative phase stability”. This comment can be of less relevance for implicit solvent CG models where the motion of lipids and already formed clusters is less hindered by solvent molecules.

Another CG model based on the top-down approach is the ELBA model which has been developed specifically for simulations of lipid self-assembled structures [15, 48]. ELBA stands for an electrostatic-based model. In this model, amine and phosphate groups of lipid head contain explicit electric charges, and glycerol and ester moieties are modeled with electric dipoles. Each solvent particle represents a single water molecule and also contains an electric dipole. ELBA model contains more beads compared to the Martini model and thus requires more expensive computations. In the both above models, an explicit solvent is necessary to stabilize lipid phases. This results in an excessive number of particles compared to our model. Reduction in the degrees of freedom due to implicit solvent improves greatly the efficiency of sampling, not only due to lower number of particles to simulate, but also because of removing friction between the particles, resulting in faster dynamics [13]. Our work shows that bottom-up derived implicit solvent CG models can successfully predict aggregated lipid structures with substantially higher computational efficiency, which became especially important at high water content.

## Conclusions

We have used systematic structure based coarse-graining to develop a solvent-free CG model of DOPE molecule with 14 beads per lipid. We investigated two sets of CG potentials derived from atomistic simulations carried out at different thermodynamics conditions corresponding to different lipid/water molar ratio, which resulted in LC and HC models of DOPE lipids. The LC model was derived from atomistic simulations at high water content with lipids gathered into lamellar bilayer-like structure, while HC model was based on atomistic simulations at low water content with lipids forming a distorted inverted hexagonal structure with elements of lamellar structure. CG potentials refinement was done initially by the Iterative Boltzmann Inversion, while the final CG potentials, which reproduce accurately distribution functions of the atomistic simulations, were generated by the Inverse Monte Carlo method.

The LC model is only able to form L_*α*_ lipid phase while cannot stabilize the H_II_ lipid phase. In contrast, the CG potentials of the HC model are able to stabilize both L_*α*_ and H_II_ lipid phases, so that depending on the lipid density, the inverted hexagonal or bilayer structures are formed in simulations started from a random mixture of DOPE molecules. The stalk-like structures were seen as an important transition state during the formation of H_II_ lipid phase. The HC model is transferable through a range of densities, producing at specific conditions different lipid phases including the inverted hexagonal, multilayers, tubular, unilamellar vesicle and micellar structures. Non-bonded potential functions, derived from radial distribution functions of the atomistic simulations, play an important role in the overall formation of the structures, while bonded potentials, especially torsion angle interactions, are effective in stabilizing the final structures. Good transferability of the HC model can be related to the fact that the lipid structures, formed in the atomistic simulation at high lipid concentration, included both lamellar and non-lamellar structural features. Despite that bottom-up derived CG models are known to be state-point dependent, CG potentials can show reasonable transferability if underlying simulations include features of different phases. Previously, similar effect was observed for systematically derived one-site water model which showed substantially improved transferability when atomistic reference simulation was done in conditions of fluid-vapour equilibria [49].

## Supporting information

S1 Fig Bonded and non-bonded distribution functions of the HC model during IBI/IMC processes. (PDF)

S2 Fig Comparison of bonded and non-bonded potentials of the LC and HC models. (PDF)

S3 Fig Results of CG simulations with exchanged bonded and non-bonded potential functions between LC and HC models. (PDF)

S4 Fig Results of simulation of 1000 CG DOPE molecules at 50 wt%, 70 wt%, and 90 wt% water contents. (PDF)

S1 Video Formation of H_II_ lipid phase of the 1000 DOPE molecule at 18 wt% water content. (MOV)

S2 Video Formation of L_*α*_ lipid phase of the 1000 DOPE molecule at 32 wt% water content. (MOV)

S1 Data Tabulated CG potentials for the LC and HC models, GROMACS setup files for the atomistic simulations, and LAMMPS script files for simulating 1000 DOPE molecules at 18 wt% and 32 wt% water content. (ZIP)
