# Supplementary material for: Implicit solvent systematic coarse-graining of dioleoylphosphatidylethanolamine lipids: From the inverted hexagonal to the bilayer structure
Source: PLoS One. 2019 Apr 5;14(4):e0214673. doi: 10.1371/journal.pone.0214673 (PMC6450619; doi:10.1371/journal.pone.0214673)
Supplement: S1 Fig — (PDF) [file pone.0214673.s001.pdf]

Bonded and non-bonded distribution functions of the HC model during IBI/IMC processes.

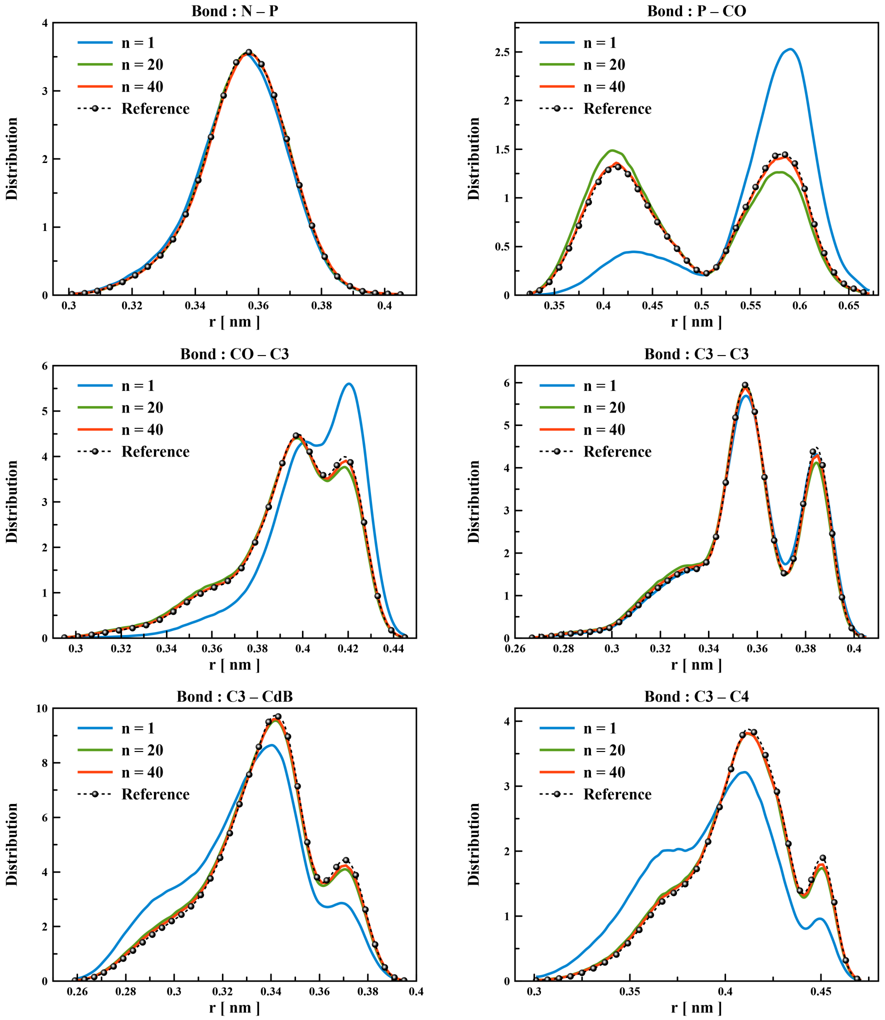


**Fig S1(A).** Bond distribution functions of the HC model during iteration (n).

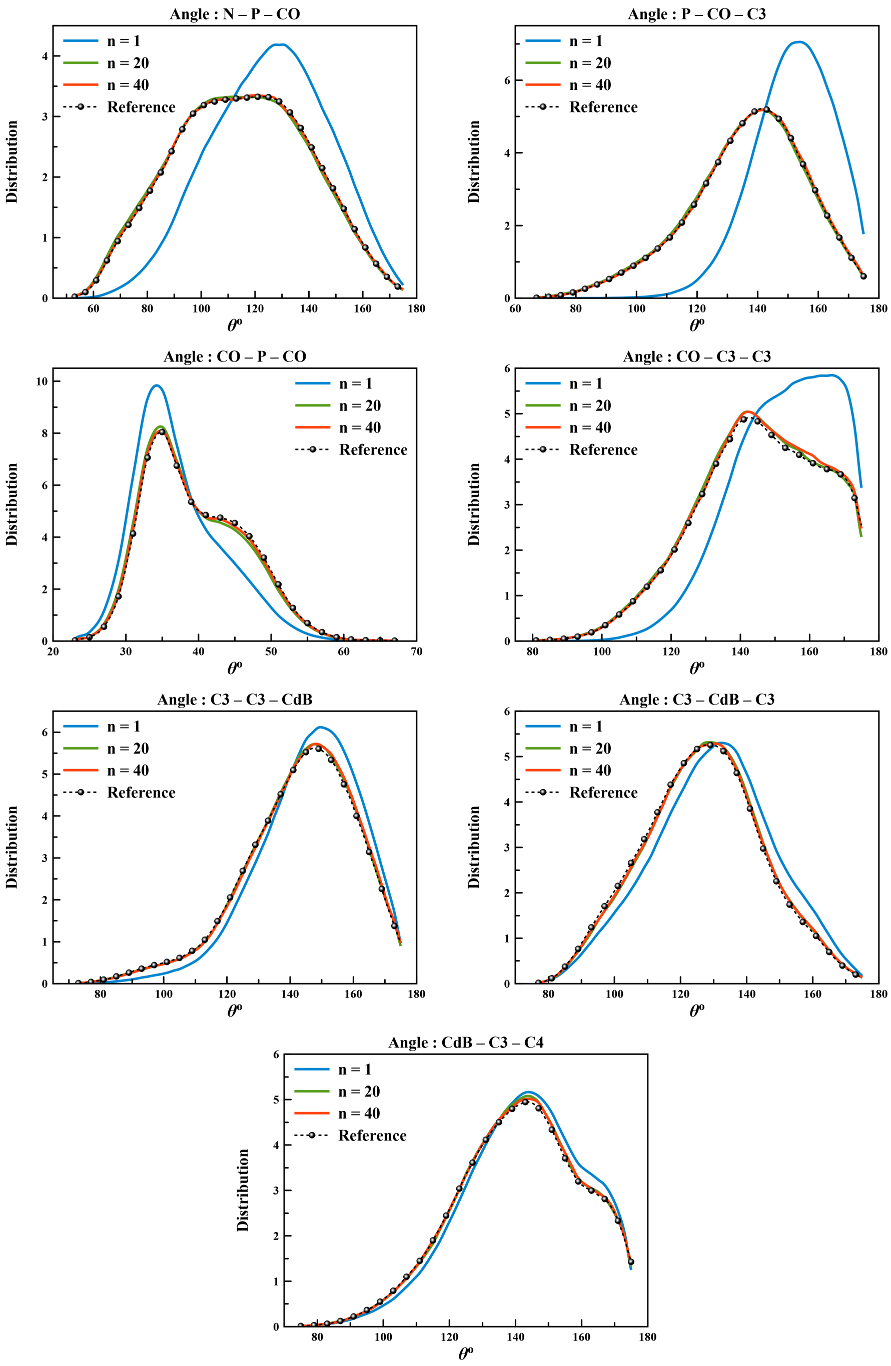


**Fig S1(B).** Angle distribution functions of the HC model during iteration (n).

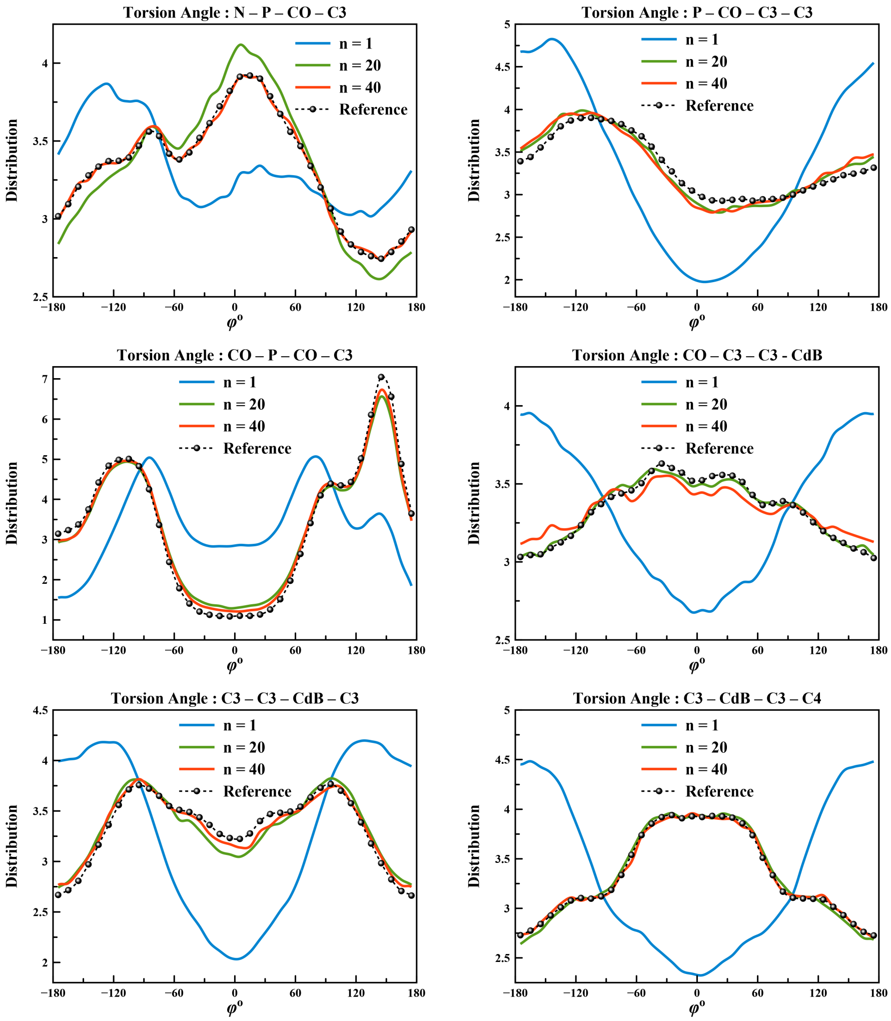


**Fig S1(C).** Torsion angle distribution functions of the HC model during iteration (n).

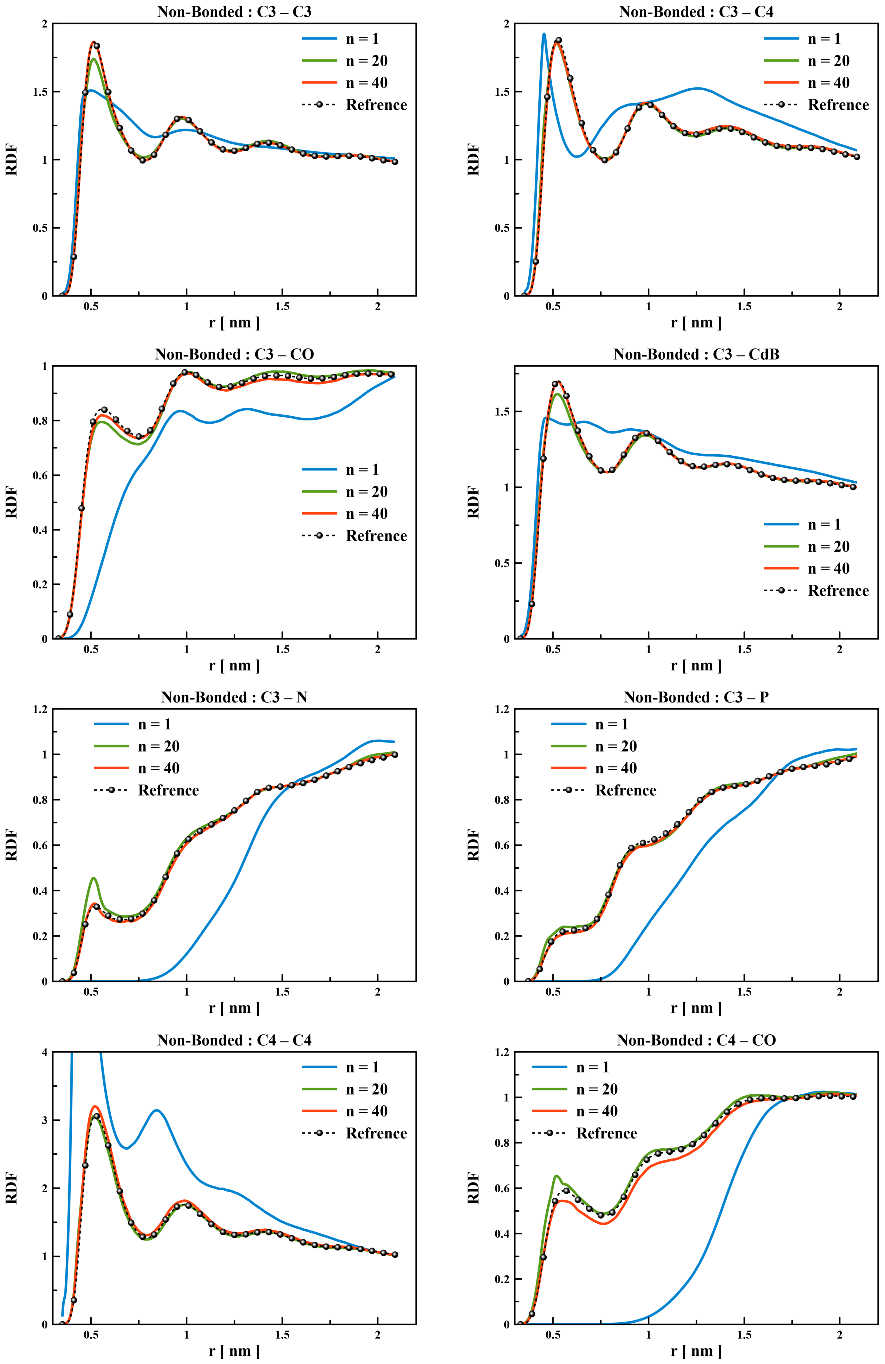


**Fig S1(D).** Radial distribution functions of the HC model during iteration (n).

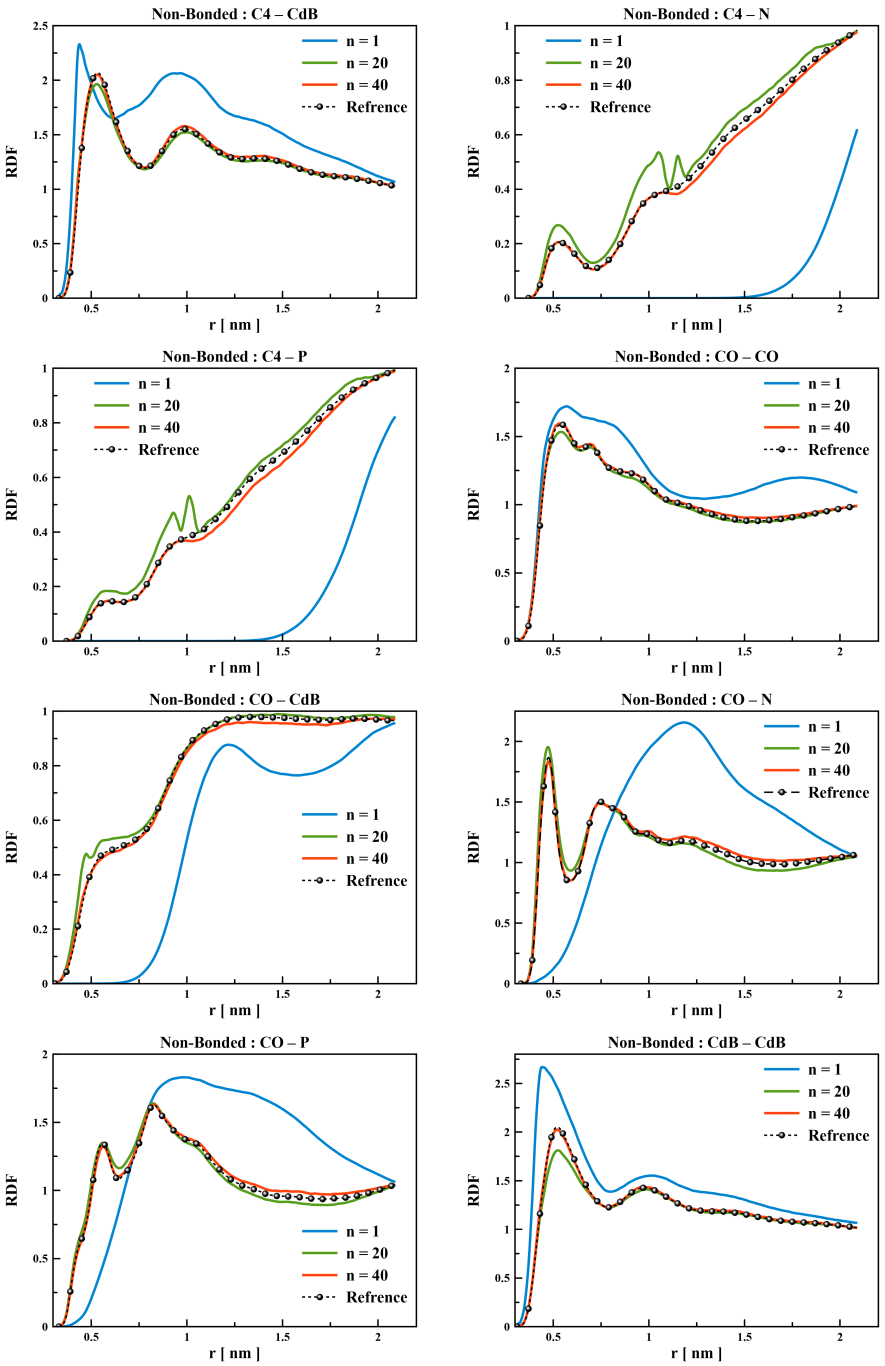


**Fig S1(E).** Radial distribution functions of the HC model during iteration (n).

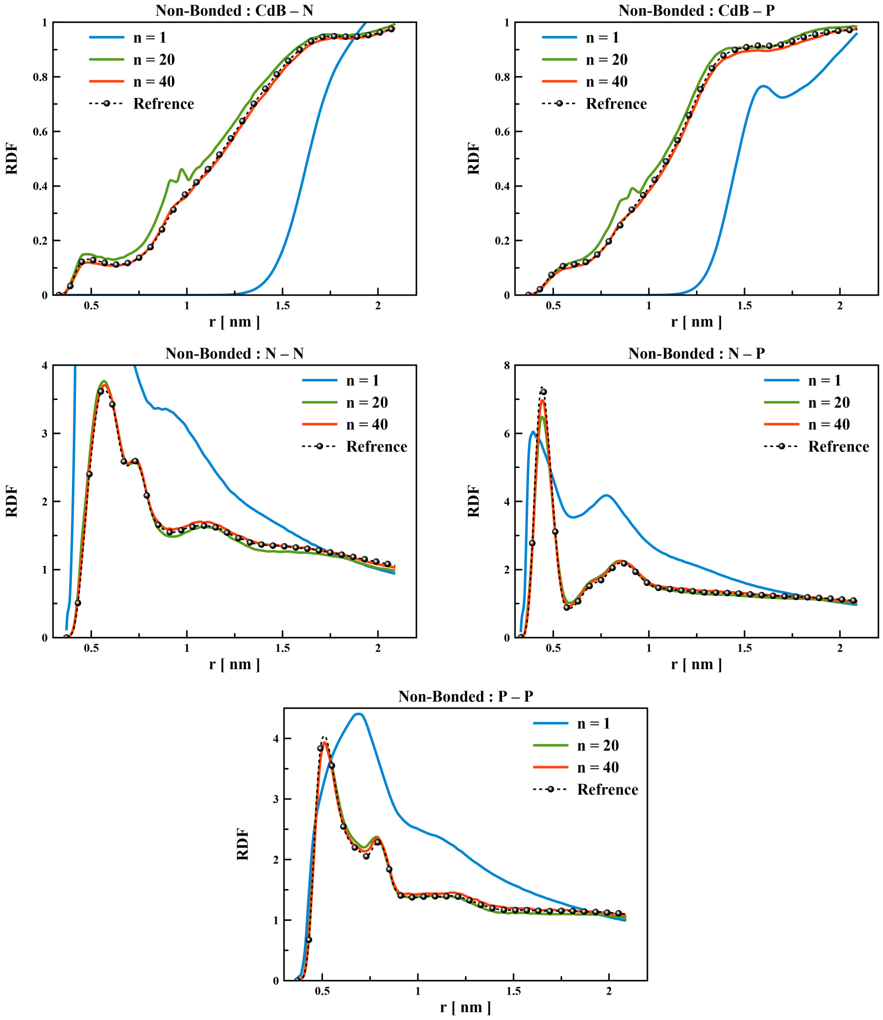


**Fig S1(F).** Radial distribution functions of the HC model during iteration (n).
