# Supplementary material for: Implicit solvent systematic coarse-graining of dioleoylphosphatidylethanolamine lipids: From the inverted hexagonal to the bilayer structure
Source: PLoS One. 2019 Apr 5;14(4):e0214673. doi: 10.1371/journal.pone.0214673 (PMC6450619; doi:10.1371/journal.pone.0214673)
Supplement: S2 Fig — (PDF) [file pone.0214673.s002.pdf]

Comparison of bonded and non-bonded potentials of the LC and HC models.

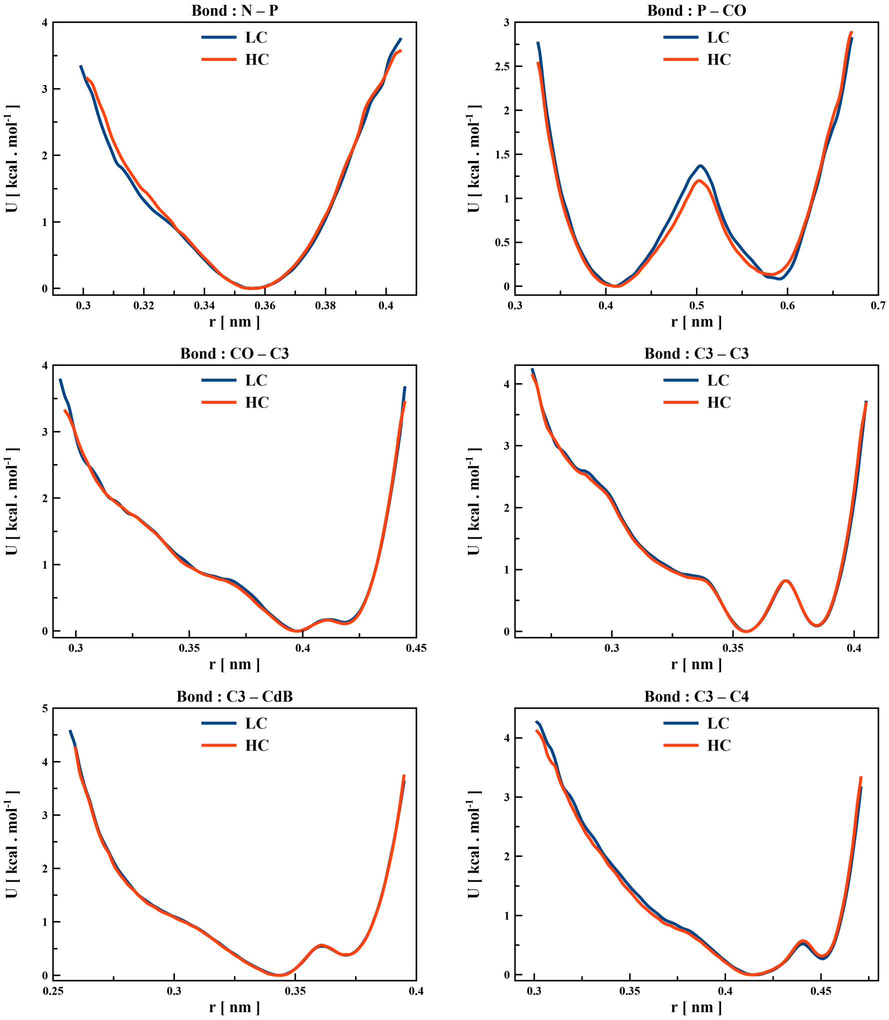

Fig S2(A). Bond potentials of the LC and HC models.

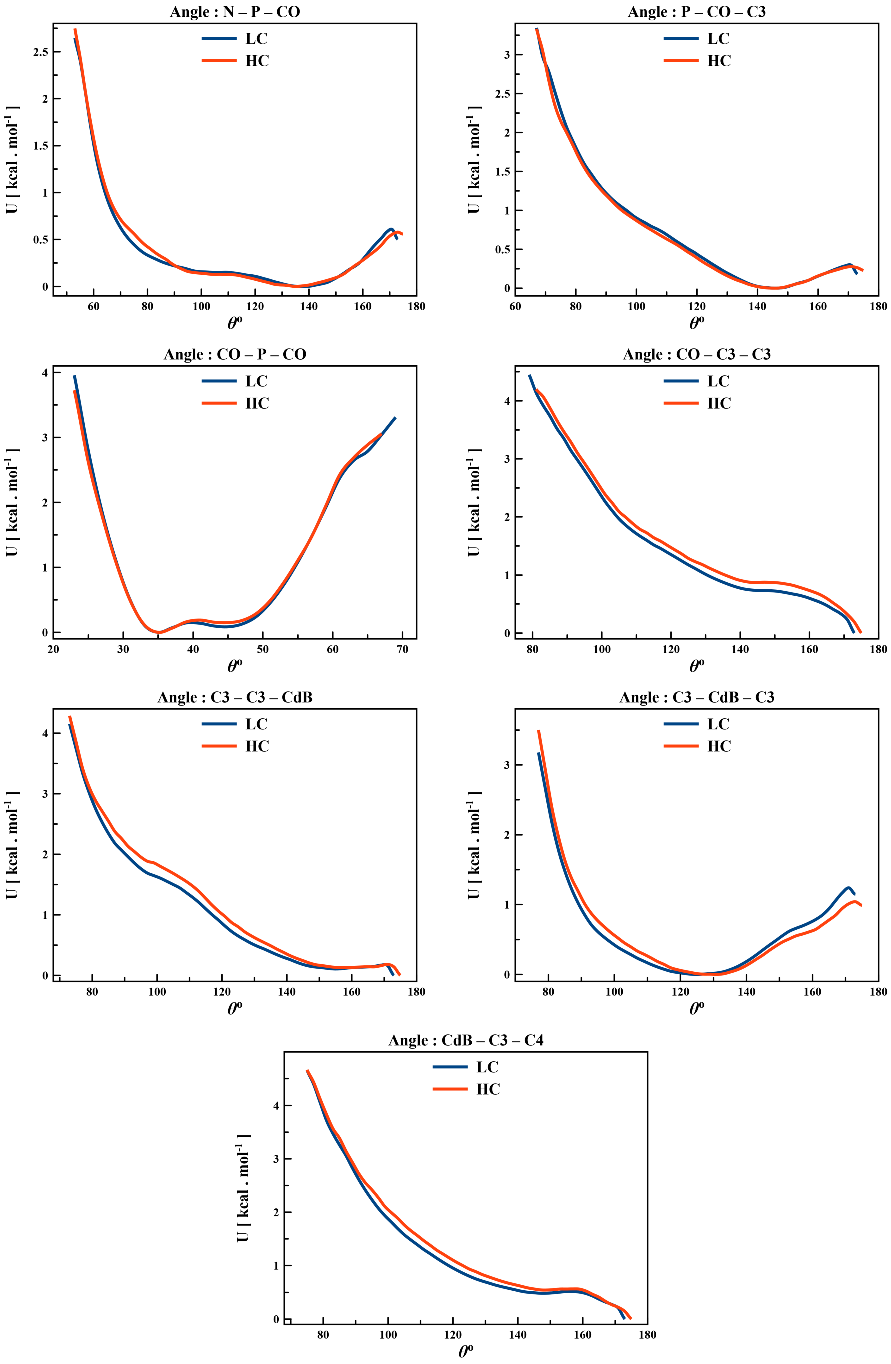

Fig S2(B). Angle potentials of the LC and HC models.

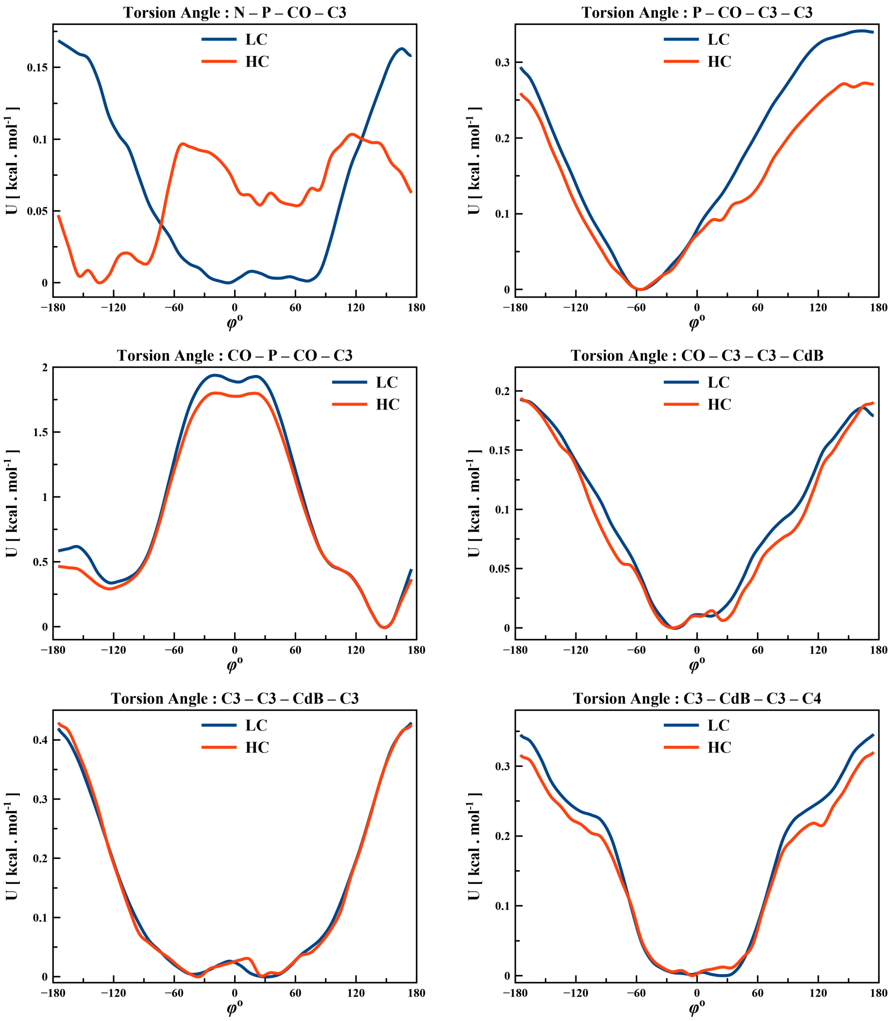

Fig S2(C). Torsion angle potentials of the LC and HC models.

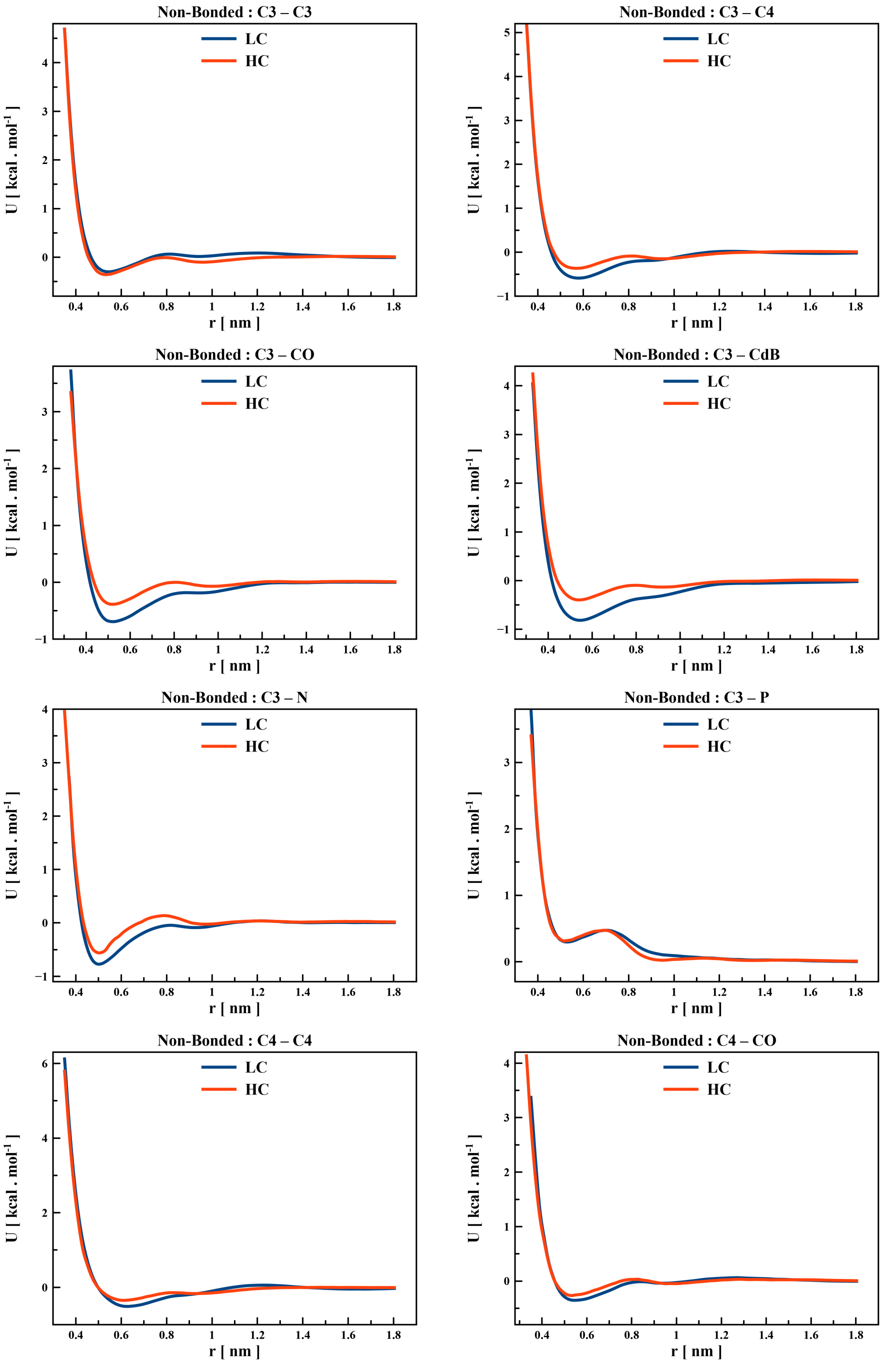

Fig S2(D). Non-bonded potentials of the LC and HC models.

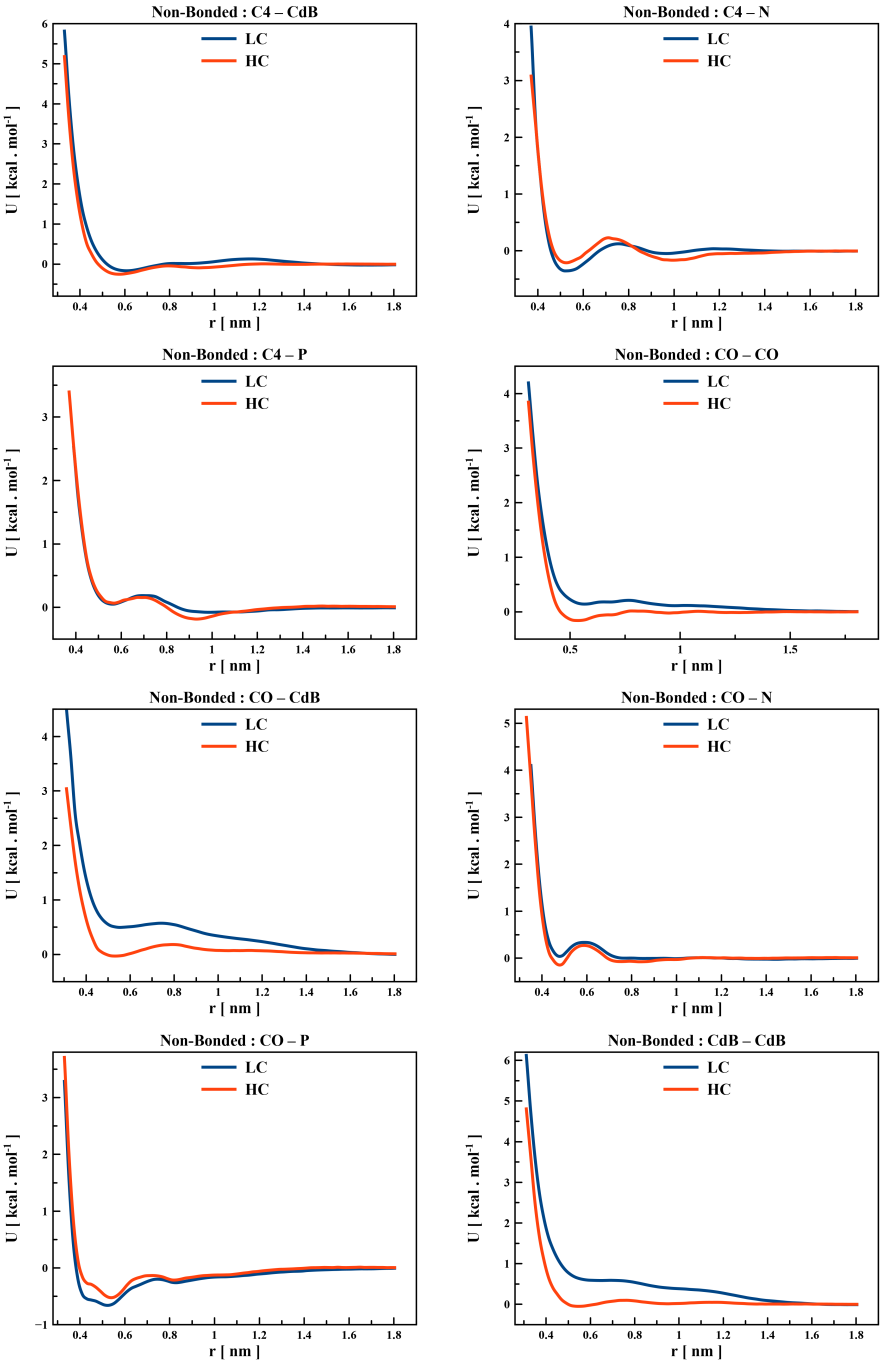

Fig S2(E). Non-bonded potentials of the LC and HC models.

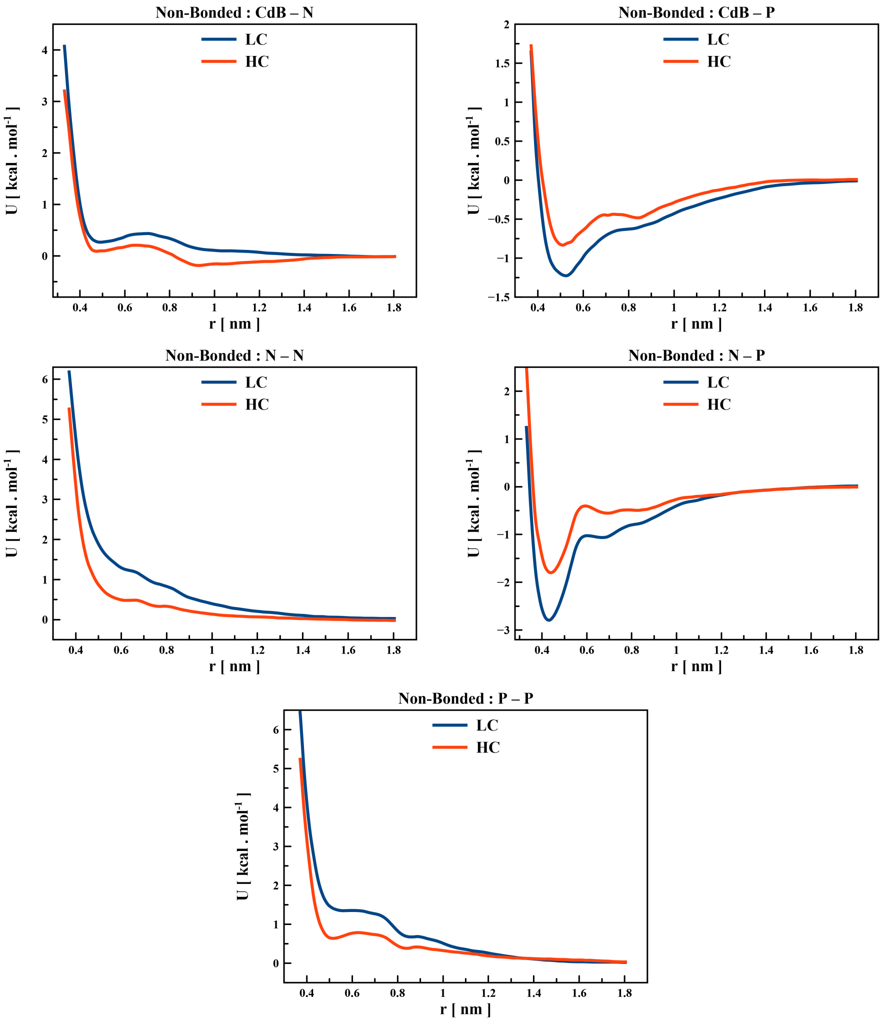

Fig S2(F). Non-bonded potentials of the LC and HC models.
