# Supplementary material for: Implicit solvent systematic coarse-graining of dioleoylphosphatidylethanolamine lipids: From the inverted hexagonal to the bilayer structure
Source: PLoS One. 2019 Apr 5;14(4):e0214673. doi: 10.1371/journal.pone.0214673 (PMC6450619; doi:10.1371/journal.pone.0214673)
Supplement: S3 Fig — (PDF) [file pone.0214673.s003.pdf]

Results of CG simulations with exchanged bonded and non-bonded potential functions between LC and HC models.

**CG Potential**	**Initial structure**	**Final structure (view 1)**	**Final structure (view 2)**
$U^{HC}_{Bonded} + U^{LC}_{Non-Bonded}$	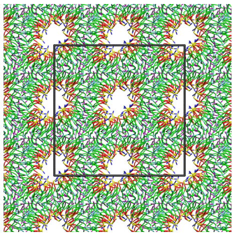	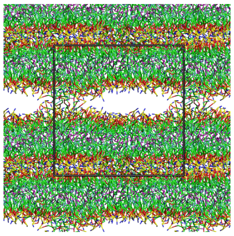	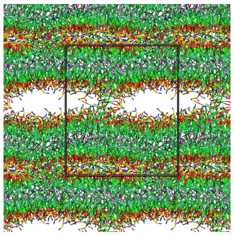
$U^{LC}_{Bonded} + U^{HC}_{Non-Bonded}$	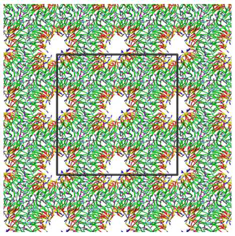	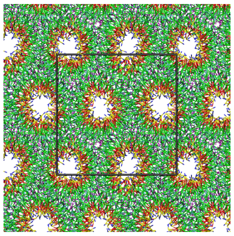	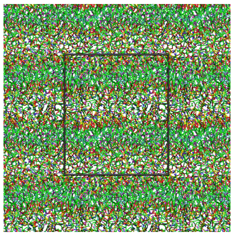
$U^{LC}_{Bonded} + U^{HC}_{Non-Bonded}$	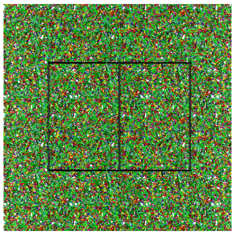	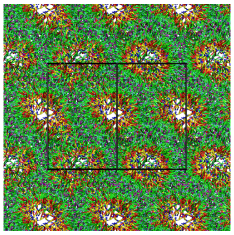	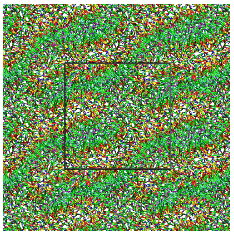

Fig S3. Simulation of 480 lipid molecules at 18 wt% water content for 400 ns using different composition of bonded and non-bonded potentials of the LC and HC models.
