# Supplementary material for: Implicit solvent systematic coarse-graining of dioleoylphosphatidylethanolamine lipids: From the inverted hexagonal to the bilayer structure
Source: PLoS One. 2019 Apr 5;14(4):e0214673. doi: 10.1371/journal.pone.0214673 (PMC6450619; doi:10.1371/journal.pone.0214673)
Supplement: S4 Fig — (PDF) [file pone.0214673.s004.pdf]

Results of simulation of 1000 CG DOPE molecules at 50 wt%, 70 wt%, and 90 wt% water contents.

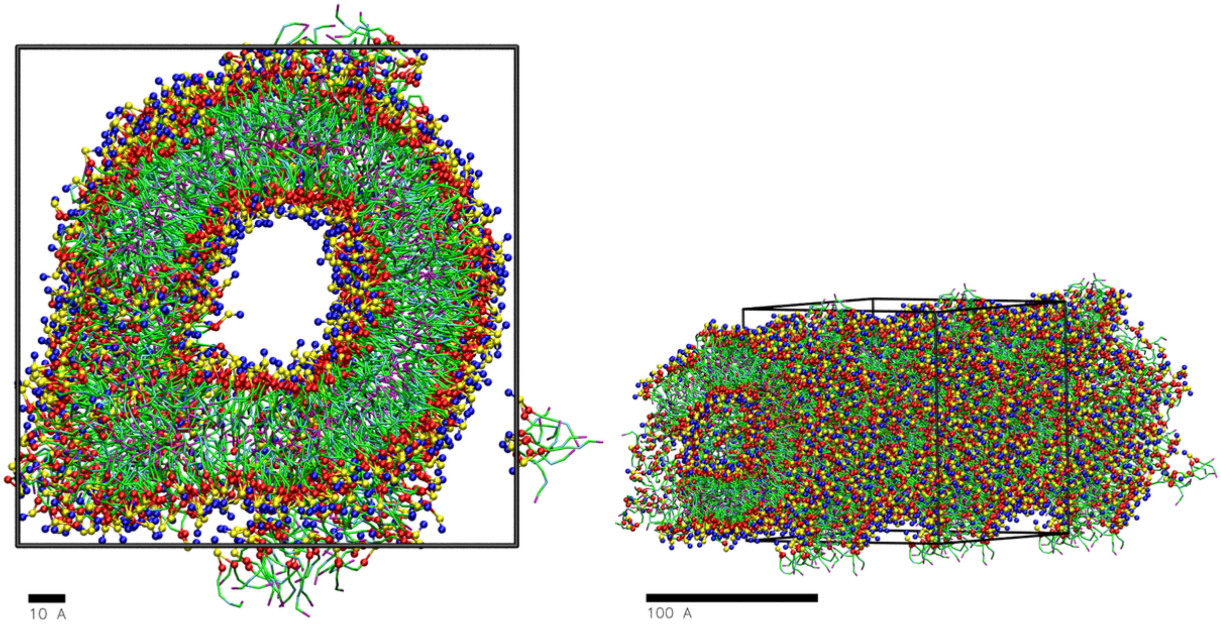

Fig S4(A). 50 wt% water content. Periodic images in the x-direction are turned on.

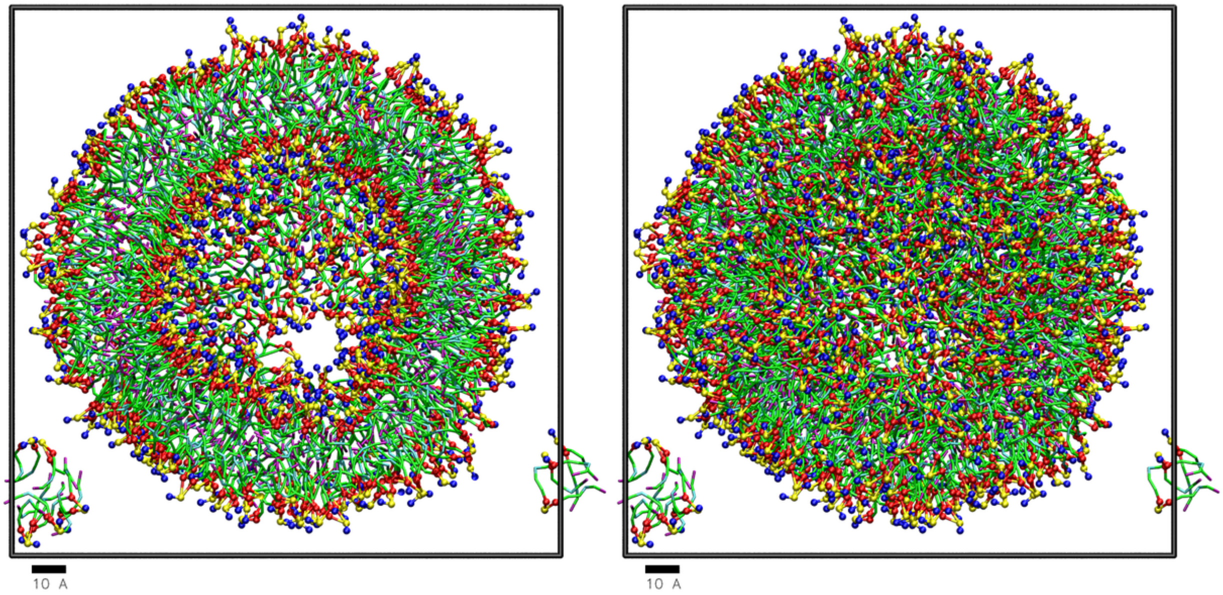

Fig S4(B). 70 wt% water content.

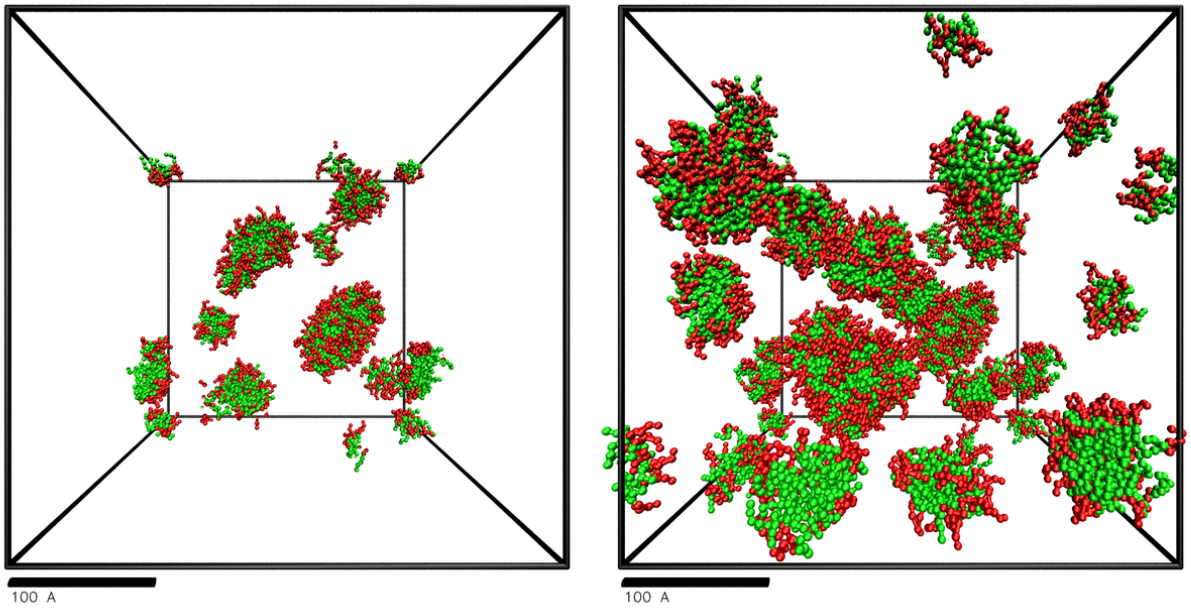

Fig S4(C). 90 wt% water content.
